# HIV-DNA Given with or without Intradermal Electroporation Is Safe and Highly Immunogenic in Healthy Swedish HIV-1 DNA/MVA Vaccinees: A Phase I Randomized Trial

**DOI:** 10.1371/journal.pone.0131748

**Published:** 2015-06-29

**Authors:** Charlotta Nilsson, Bo Hejdeman, Karina Godoy-Ramirez, Teghesti Tecleab, Gabriella Scarlatti, Andreas Bråve, Patricia L. Earl, Richard R. Stout, Merlin L. Robb, Robin J. Shattock, Gunnel Biberfeld, Eric Sandström, Britta Wahren

**Affiliations:** 1 Department of Microbiology, Public Health Agency of Sweden, Solna, Sweden; 2 Department of Microbiology, Tumor and Cell biology, Karolinska Institutet, Stockholm, Sweden; 3 Department of Laboratory Medicine, Karolinska Institutet, Huddinge, Sweden; 4 Venhälsan, Department of Education and Clinical Research, Karolinska Institutet, Södersjukhuset, Stockholm, Sweden; 5 Viral Evolution and Transmission Unit, Division of Immunology, Transplantation and infectious diseases, IRCCS San Raffaele Scientific Institute, Milan, Italy; 6 National Institute of Allergy and Infectious Diseases, National Institutes of Health, Bethesda MD, United States of America; 7 Bioject Inc., Tigard, Oregon, United States of America; 8 Military HIV Research Program, Walter Reed Army Institute of Research, Rockville, Maryland, United States of America; 9 Imperial College London, Department of Infectious Diseases, Division of Medicine, Norfolk Place, London, United Kingdom; Asociacion Civil Impacta Salud y Educacion, PERU

## Abstract

**Background:**

We compared safety and immunogenicity of intradermal (ID) vaccination with and without electroporation (EP) in a phase I randomized placebo-controlled trial of an HIV-DNA prime HIV-MVA boost vaccine in healthy Swedish volunteers.

**Methods:**

HIV-DNA plasmids encoding HIV-1 genes gp160 subtypes A, B and C; Rev B; Gag A and B and RTmut B were given ID at weeks 0, 6 and 12 in a dose of 0.6 mg. Twenty-five volunteers received vaccine using a needle-free device (ZetaJet) with (n=16) or without (n=9) ID EP (Dermavax). Five volunteers were placebo recipients. Boosting with recombinant MVA-CMDR expressing HIV-1 Env, Gag, Pol of CRF01_AE (HIV-MVA) or placebo was performed at weeks 24 and 40. Nine of the vaccinees received a subtype C CN54 gp140 protein boost together with HIV-MVA.

**Results:**

The ID/EP delivery was very well tolerated. After three HIV-DNA immunizations, no statistically significant difference was seen in the IFN-γ ELISpot response rate to Gag between HIV-DNA ID/EP recipients (5/15, 33%) and HIV-DNA ID recipients (1/7, 14%, p=0.6158). The first HIV-MVA or HIV-MVA+gp140 vaccination increased the IFN-γ ELISpot response rate to 18/19 (95%). CD4^+^ and/or CD8^+^ T cell responses to Gag or Env were demonstrable in 94% of vaccinees. A balanced CD4^+^ and CD8^+^ T cell response was noted, with 78% and 71% responders, respectively. IFN-γ and IL-2 dominated the CD4+ T cell response to Gag and Env. The CD8^+^ response to Gag was broader with expression of IFN-γ, IL-2, MIP-1β and/or CD107. No differences were seen between DNA vaccine groups. Binding antibodies were induced after the second HIV-MVA+/-gp140 in 93% of vaccinees to subtype C Env, with the highest titers among EP/gp140 recipients.

**Conclusion:**

Intradermal electroporation of HIV-DNA was well tolerated. Strong cell- and antibody-mediated immune responses were elicited by the HIV-DNA prime and HIV-MVA boosting regimen, with or without intradermal electroporation use.

**Trial Registration:**

International Standard Randomised Controlled Trial Number (ISRCTN) 60284968

## Introduction

Globally in 2012, UNAIDS estimated that 35.3 (32.2–38.8) million people were living with HIV. The number of new infections declined 33% since 2001 to 2.3 (1.9–2.7) million in 2012. The number of AIDS deaths had also declined to 1.6 (1.4–1.9) million in 2012 from 2.3 (2.1–2.6) million in 2005. These changes reflect an increased access of antiretroviral therapy globally. However, HIV antiretroviral treatment coverage in low-and middle-income countries amounted to only 34% (32–37%) of the 26.8 million eligible for treatment in 2013 [1]. Notwithstanding increased intervention efforts, a safe and effective HIV vaccine is still needed [2,3]. The ideal HIV vaccine would prevent acquisition of infection and control viral replication.

Pre-clinical studies in nonhuman primates and studies in HIV-infected individuals have suggested that both cell-mediated and antibody-mediated virus-specific immune responses would contribute to the efficacy of an HIV vaccine. Simian immunodeficiency virus (SIV) Env-specific antibodies exhibiting binding and functional properties were suggested to be critical for protection from virus acquisition in monkeys [4]. CD8^+^ T cells can mediate control of viral replication in HIV-infected individuals and SIV-infected monkeys [5]. Furthermore, cytolytic CD4^+^ T cells were shown to be important in both early viral control in acute HIV infection [6] and in attenuated SIV infection [7].

HIV-vaccine efficacy in humans was demonstrated in the RV144 trial in 2009. A canary pox (ALVAC) prime and an envelope protein (AIDSVAX) boost vaccine induced a modest 31.2% reduction of transmission in low-incidence Thai heterosexuals, without effects on HIV viral load or CD4^+^ T cell counts in infected individuals [8]. Ensuing immune-correlates analyses demonstrated that binding IgG antibodies to variable regions 1 and 2 (V1V2) of HIV-1 envelope proteins [9–11] and IgG to linear epitopes in the V2 and V3 regions correlated inversely with the risk of HIV infection [12]. Analysis of the T cell responses in the RV144 vaccinees confirmed HIV gp120 V2 specificity and revealed CD4^+^ T cells exhibiting polyfunctionality and cytolytic capacity [13].

Several HIV prime-boost vaccine concepts are being tested in phase I/II trials [3]. In the majority of these clinical trials, plasmid DNA is being used for priming immunizations. The DNA vaccine is followed by boosting immunizations using either, envelope protein, a recombinant pox virus vector or a recombinant adenovirus vector. DNA vaccines have advantages over other vaccines since they allow for repeat administration due to lack of anti-vector responses, the focusing of immune response, stability over a wide range of storage conditions and their excellent safety profiles [14]. DNA vaccines on their own have been poorly immunogenic in humans. Nonetheless, we and others have shown that they are an effective priming modality in clinical HIV prime boost vaccine regimens [15–23].

Strategies to increase plasmid DNA immunogenicity are being sought. In vivo electroporation (EP) is one such strategy that is being explored. EP enhances DNA uptake efficacy by the transient formation of pores in the cell membrane. It also results in an influx of APCs and other immune cells to the site of injection and causes a local inflammatory reaction, which will further enhance the immunogenicity of the gene product [24–26]. Non-human primate studies have shown that intramuscular EP increases the immunogenicity of SIV DNA vaccines [27, 28]. Two phase I HIV vaccine trials using intramuscular EP have been performed. Findings in both trials show enhanced cellular immune responses when applying intramuscular EP for DNA vaccine delivery [29, 30]. However, there was considerable discomfort associated with the intramuscular EP [29]. Since ID EP has been shown to be very effective in preclinical studies [31,32,33], it might offer a more acceptable alternative, if shown to be effective also in humans.

In the present hypothesis-generating phase I clinical trial in Stockholm, Sweden we determined the safety, tolerability and immunogenicity of an HIV-DNA prime HIV-MVA boost vaccine regimen. The priming plasmid DNA vaccine was delivered intradermally (ID) using a needle free device (ZetaJet) with or without intradermal EP applied with the Dermavax (Cellectis) device. Boosting vaccinations were performed with recombinant modified vaccinia virus Ankara (MVA) expressing HIV-1 genes. A group of vaccinees was randomized to receive CN54 subtype C gp140 protein (without adjuvant) at the time of the HIV-MVA vaccinations in order to obtain preliminary toxicity and immunogenicity data.

## Material and Methods

### Ethics statement

The protocols and products were approved by Regionala Etikprövningsnämnden i Stockholm (Regional Ethics Committee in Stockholm), and the Swedish Medical Products Agency. Written informed consent was obtained from all trial participants.

### Study design

Thirty healthy HIV-1 negative Swedish volunteers were recruited into a phase I double-blind placebo-controlled clinical trial. The trial is registered in the International Standard Randomised Controlled Trial Number (ISRCTN) Register with registration number 60284968 and can be accessed at http://www.controlled-trials.com/ISRCTN60284968, doi 10.1186/ISRCTN60284968. The study protocol and CONSORT checklist are available as supporting documents; see S1 Protocol and S1 CONSORT Checklist. The first volunteer was enrolled October 20, 2010 and the last follow-up visit was completed March 13, 2013. The trial aimed to recruit 36 participants, but was restricted to 30 participants due to recruitment difficulties (low remuneration and many visits) after consultation with the Medical Products Agency.

The volunteers were randomized into three different treatment arms (Table 1) to be injected three times with 0.6 mg of HIV-1 DNA plasmids or placebo intradermally (ID) given by a needle-free spring-driven ZetaJet device (Bioject Medical Technologies, Inc., Tualatin, OR, USA) followed by intramuscular (IM) boost injections with 10^8^ pfu of HIV-MVA or placebo at weeks 24 and 40. Group 1 received ‘standard’ immunization and the other two groups of volunteers received additional intradermal electroporation performed with the Dermavax device (Cellectis AS, Romainville, France) after each immunization with HIV-1 DNA or placebo. The randomization was performed by an independent consultant to the pharmacy, APL, in two steps. First an equal number of men and women were randomized to three groups by the computer program 'Design' from two blocks of 18. In each subgroup of 6 a single placebo was randomly assigned. The remainder in each group were allocated to vaccine. The allocation was kept in the pharmacy and only the group allocation was communicated to the clinic upon a written request for an enrolment. The laboratory was blinded to both group and placebo. Electroporation was performed by pressing an array of 2 rows of 6 needle electrodes of 2 mm in length spaced 4 mm apart against the skin on the outside of the upper arms, straddling the injection site, followed by two 0.05 millisecond pulses of 450 V and eight 10 millisecond pulses of 110 V during 0.3 seconds [33]. One group of vaccinees who received DNA with EP also received 100 μg of gp140 given IM in parallel with the HIV-MVA vaccinations in the opposite arm. All participants were recruited, immunized and followed at Venhälsan, Södersjukhuset, Stockholm, Sweden.

**Table 1:** Vaccination groups and allocation.

Group	N (N)*	Mode of HIV-DNA delivery All ID ZetaJet	Left arm (Ampoules 1 Env) (w 0, 6, 12)	Right arm (Ampoules 2 Gag, RTmut) (w 0, 6, 12)	Left arm IM immunization using needle (w 24 and 40)
1	10 (9)	No electroporation	1 inj 100 μl 0.3 mg DNA	1 inj 100 μl 0.3 mg DNA	1 inj HIV-MVA 10^8^ pfu (1.0 ml)
	2 (2)	No electroporation	1 inj 100 μl saline	1 inj 100 μl saline	1 inj 1.0 ml saline
2	10 (7)	Electroporation	1 inj 100 μl 0.3 mg DNA	1 inj 100 μl 0.3 mg DNA	1 inj HIV-MVA 10^8^ pfu (1.0 ml)
	2 (2)	Electroporation	1 inj 100 μl saline	1 inj 100 μl saline	1 inj 1.0 ml saline
3	10 (9)	Electroporation	1 inj 100 μl 0.3 mg DNA	1 inj 100 μl 0.3 mg DNA	1 inj HIV-MVA 10^8^ pfu (1.0 ml) + 100 μg (0.2 ml) gp140
	2 (1)	Electroporation	1 inj100 μl saline	1 inj100 μl saline	1 inj 1.0 ml + 0.2 ml saline

*Actual number of volunteers randomized in the vaccination groups.

### Vaccines

The vaccinees were immunized with HIV-DNA plasmids encoding envelope (Env) gp160 of HIV-1 subtypes A, B and C; Gag of subtypes A and B and reverse transcriptase (RT) and Rev of subtype B, which has been detailed elsewhere [34]. The seven DNA plasmids were delivered as two entities; one containing the Gag- and RT-encoding plasmids, the other containing the Env- and Rev-encoding plasmids. The DNA vaccine was produced by Vecura, Karolinska University Hospital, Huddinge, Sweden. The HIV-DNA immunizations were followed by two boosts with recombinant modified vaccinia virus Ankara (MVA)-Chiang Mai double recombinant (CMDR) encoding HIV-1 Env of subtype E and Gag-Pol of subtype A from Thai isolates CM235 and CM240, respectively (HIV-MVA) [35]. The vaccine was produced by the US Military HIV Research Program, Forest Glen, MD, USA. One group of vaccinees received in addition an immunization of Env gp140 of subtype C without adjuvant. The gp140 clade C envelope (gp120 plus the external domain (ED) of gp41), designated CN54gp140, was produced as a recombinant product in CHO cells (S. Jeffs personal communication), and the protein was manufactured to GMP specification by Polymun Scientific (Vienna, Austria). The Env gp140 protein is encoded by the CN54gp140REKR HIV-1 envelope gene cassette derived from the clade-C/B’ HIV-1 molecular clone p97CN54 of Chinese origin [36].

### Safety

Safety evaluations, which included physical examinations and laboratory tests, were performed before and 2 weeks after each vaccination, and 3 months after the last vaccination. A 12-lead electrocardiogram (ECG) was performed before and 2 weeks after the HIV-MVA vaccinations. A diary card was completed by each volunteer for 7 days following each vaccination to record local and systemic adverse events, as well as medication taken. Volunteers were also contacted the day following injections for a brief interview about possible adverse reactions. Subjects reporting any grade 2 or higher event were referred for a clinic visit.

Safety laboratory tests included a complete blood count, aspartate aminotransferase (AST), alanine aminotransferase (ALT), total bilirubin, creatinine, fasting blood glucose and complete urine analysis including pregnancy test for female participants. The hematology and biochemistry tests were performed at Karolinska University Hospital, Huddinge, Sweden. T-lymphocyte subset determinations were performed using FACSCalibur (Becton Dickinson, NJ, USA).

Diagnostic HIV serological testing was performed using Architect HIV Ag/Ab Combo (Abbott Diagnostics, Wiesbaden, Germany), which is a chemiluminescent microparticle immunoassay. Infection status was determined by HIV RNA detection using the Roche Amplicor HIV-1 Monitor v1.5 RNA-PCR kit (Roche Diagnostic Systems).

### Blood collection and cell preparation

Whole-blood samples for analysis of immune responses were collected in cell preparation tubes (CPT Vacutainer tubes, BD) or standard Vacutainer tubes (BD). Both contained sodium heparin as anticoagulant. PBMCs from the latter were purified using Leucosep tubes (Greiner Bio-One GmbH, Frickenhausen, Germany). PBMCs were processed and stored as described previously [37].

### ELISpot assay

Interferon gamma (IFN-γ) ELISpot was performed on fresh PBMCs using the h-IFN-γ ELISpot^PLUS^ kit and a 2-step detection system according to the manufacturer’s instructions (Mabtech, Nacka, Sweden) as described previously [16]. Peptide pools specific for the HIV-DNA vaccine [16] and the HIV-MVA vaccine, here designated Gag CMDR, Env CMDR and Pol CMDR, were used for stimulation (S1 Table).

### T cell proliferation assay

A tritiated [^3^H]-thymidine uptake lymphoproliferation assay was used as reported previously [38]. Aldrithiol-2 (AT-2) treated HIV-1_MN_ (subtype B isolate) and HIV-1_CM235_ (CRF01_AE isolate) and SUPT1 microvesicles or Jurkat Tat-CCR5 microvesicles (control antigens) were used for stimulation. The antigens were kindly provided by Dr. J. Lifson, SAIC Frederick, Inc., Frederick, USA.

### 8-colour ICS assay

For the determination of CD4^+^ and CD8^+^ T cell responses, an 8-colour ICS assay was performed on cryopreserved PBMCs. The PBMCs were thawed and rested overnight at 37°C in a 7.5% CO_2_ incubator. The next day cells were counted and the cell concentration was adjusted. Stimulation was performed in the presence of anti-CD28 and anti CD49 (BD) at final concentrations of 1 μg/ml, monensin (BD) at 1 μg/ml and Brefeldin A at 5 mg/ml (Sigma-Aldrich, Germany). The cells were incubated for 6 hours with HIV-specific peptide pools. The staining procedures were described previously [39]. Briefly, surface staining was performed using anti-CD4-V500, anti-CD8-PerCPCy5.5, anti-CD3–APC-H7 (Becton Dickinson, San Jose, CA) and the viability marker VIVID-Pacific blue (Life Technologies), while intra-cellular staining was performed using anti-MIP-1β-PE, anti-IFN-γ-FITC, anti-IL-2-PE-Cy7 and anti-CD107a-APC (Becton Dickinson, San Jose, CA). Anti-CD107a-APC was added to the cell suspension at the start of the stimulation as described by Betts et al. [40]. Acquisition of samples was performed using a FACSCanto II flow cytometer (BD Biosciences). The samples were analyzed using FlowJo software, version 8.8.2 (Tree Star, Ashland, OR) and distributions were presented using PESTLE and SPICE, version 5.3 (kindly provided by Mario Roederer, Vaccine Research Center, NIH, USA at http://exon.niaid.nih.gov/spice).

### ELISA for Env-binding antibodies

Antibodies to native gp160 subtype B (HIV-1_IIIB_, Advanced Biotechnologies Inc, Columbia, MD, USA) and recombinant gp140 subtype C (HIV-1_96ZM651_, kindly provided by Programme EVA, Centre for AIDS Reagents, NIBSC, Potters Bar, UK) were determined by means of standard validated ELISAs [39].

### Assays for determination of neutralizing antibodies

Neutralizing antibodies were measured in both the TZM-bl and the PBMC neutralization assay platforms as described previously [41].

For the PBMC-based assay, six steps of 2-fold dilution, starting with 1:20 of each serum, were incubated with 20 and 40 TCID50 of viral supernatant of SF162 (subtype B) and CM244 (CRF01_AE), respectively for 1 hour. Subsequently, 10^5^ PHA-stimulated PBMC were added, cells were washed at days 1 and 3 and at day 7 each well was tested for the presence of p24-antigen in an in-house ELISA (www.aaltobioreagents.ie). Neutralization titers are defined as the reciprocal of the highest serum dilution giving at least a 90% reduction of HIV-1 p24-antigen compared to virus control.

For the TZM-bl-assay, SF162 (subtype B) pseudotyped virus (PSV) and 93MW965.26 (subtype C) PSV were used. Briefly, four steps of 3-fold dilution, starting with 1:20 of each serum, were incubated with viral supernatant (200 TCID_50_) for 1 hour. Thereafter, 10^4^ TZM-bl cells were added and plates incubated for 48h, when luciferase activity was measured. Neutralization titers were defined as the sample dilution at which relative luminescence units (RLU) were reduced by 50% compared to virus control wells after subtraction of background RLU in control wells with only cells.

### Data analysis

Since this was planned as a phase I study it was judged that 10 volunteers per group would give an indication of the safety and tolerability without exposing too many participants to the interventions. Clinical and safety laboratory data were entered in the computerized hospital patient registry system under the national identification code and full name. Study data were entered under study code and initials on clinical report forms. Specimens for immunological and virological studies were sent under study code to the Swedish Institute for Communicable Disease Control, which as of January 2014 is The Public Health Agency of Sweden, from where samples were distributed to IRCCS San Raffaele Scientific Institute, Milan, Italy. The laboratories remained blinded to the randomization throughout the trial. The primary endpoint was safety and tolerability. This was assessed with standardized visual analogue scales (VAS) up to 30 minutes post injections, in a diary card during the first 7 days and with solicited adverse events and routine laboratory assays at 2 weeks post injections. Any grade 3 event was taken to indicate that the treatment is less tolerable which would lead to a study halt. Volunteer data and immunological data were analysed in SPSS 15.0 under study code. The secondary endpoint was IFN-γ ELISpot reactivity 2 weeks after the last HIV-DNA immunization and after the HIV-MVA boosts. Group 1 served as the control group and corresponded to historical data. The addition of electroporation was assessed in groups 2 and 3. Group 3 was a descriptive assessment of additional gp140 given together with the HIV-MVA boost. Most data are presented without statistical analysis since this is a descriptive, hypothesis-generating study. The magnitudes of immune responses were compared using Mann-Whitney U-test. Fischer’s Exact test was used for comparisons between groups. A p value of <0.05 was considered statistically significant.

## Results

### Enrollment

**Fig 1:**
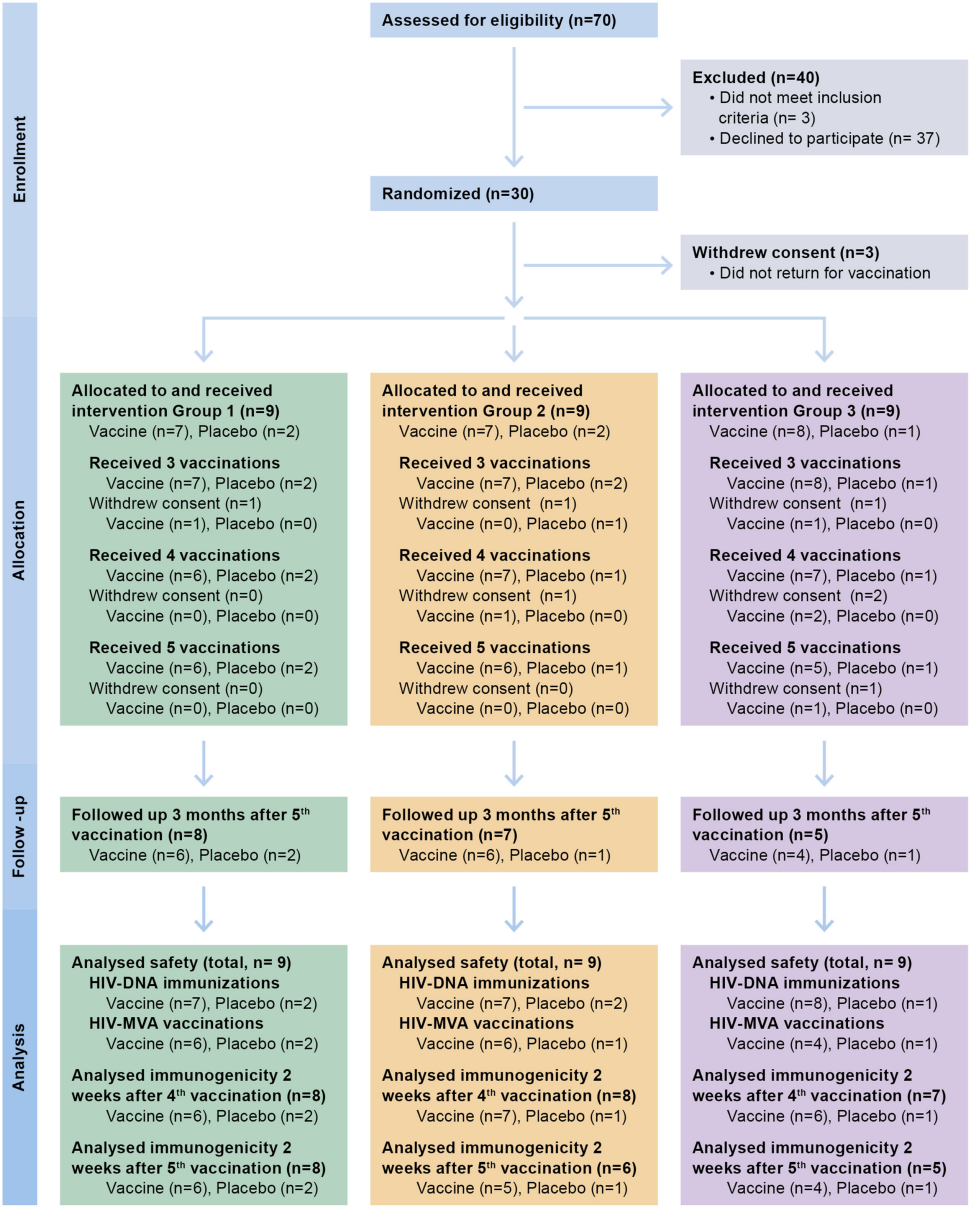
Flow diagram showing the number of individuals enrolled, randomized to vaccine or placebo, followed-up and analysed.

A total of 275 individuals were contacted by telephone for possible participation. Seventy individuals came for the first screening visit. Thirty-seven individuals declined participation, mainly due to social concerns. Thirty-three volunteers came for screening visit 2 of whom three volunteers did not satisfy the inclusion criteria. Thirty volunteers were randomized but only 27 (14 men and 13 women) started vaccinations and completed all three HIV-DNA/placebo immunizations. The first HIV-MVA/placebo vaccination was given to 24 volunteers and 21 completed two HIV-MVA/placebo vaccinations. Twenty volunteers came for the last follow-up visit 3 months after the second HIV-MVA/placebo vaccination (Fig 1). The reasons for dropping out were related to social factors and not associated with the immunization procedure or adverse events. Demographics for the trial participants are shown in Table 2 by group and overall.

**Table 2:** Baseline characteristics of participants.

	**Group 1**	**Group 2**	**Group 3**	**All placebo**	**Total**
****Female****	4	3	4	2	13
****Male****	3	4	4	3	14
****Age (years)****	31.7 (+/-5.2)	25.6 (+/-4.7)	30.6 (+/-6.4)	30.8 (+/-9)	30.0 (+/-6.4)
****Height (cm)****	174.6 (+/-11)	178.1 (+/-6.6)	171.4 (+/-10)	174.4 (+/-6)	174.5 (+/-8.8)
****Weight (kg)****	78 (+/-19.7)	82 (+/-13.5)	71 (+/-18.7)	82 (+/-20)	78 (+/-17.6)

### Safety and tolerability

In general the vaccinations and immunization procedures were very well tolerated. No severe adverse events (grades 3 or 4) at the injection site or systemically that were judged to be probably or possibly related to the vaccinations were recorded. The tolerability of the electroporation procedure was measured by a visual pain scale (VAS) with 0 indicating no pain and 10 indicating severe pain. The median value for VAS was 3 immediately after the electroporation and 0 thirty minutes later (Table 3).

**Table 3:** Discomfort from injections with HIV-DNA using ZetaJet ID, electroporation ID or HIV-MVA IM injections according to a Visual Analogue Scale 0–10; 0 = no pain.

	**ZetaJet**		**Electroporation** ^a^		**HIV-MVA**	
****Number of volunteers****	27		18		24	
****Time from immunization****	**0 min**	**30 min**	**0 min**	**30 min**	**0 min**	**30 min**
****Number of observations****	81	81	54	54	44	44
****Median (10–90 percentile)****	2 (0–4)	0 (0–1)	3 (2–7)	0 (0–1)	1 (0–4)	0 (0–1)

^a^Observation after combination of needle-free ZetaJet delivery and electroporation

### Cell-mediated immune responses

#### IFN-γ ELISpot

The IFN-γ ELISpot assay was performed on fresh PBMCs before vaccination, 2 weeks after the third HIV-DNA/placebo immunization and 2 weeks after the first and second HIV-MVA/gp140/placebo vaccinations. None of the vaccinees had a positive response to the HIV-specific peptide pools before vaccination and none of the five placebos were ELISpot reactive at any of the time points tested. Two weeks after the third HIV-DNA immunization, 6/22 (27%) had a positive IFN-γ ELISpot to ≥ 1 HIV Gag-specific peptide pool. The response rates to Gag were 1/7 (14%), 2/7 (28%) and 3/8 (37%), respectively for vaccinees in group 1 (receiving HIV-DNA with ZetaJet but without EP), group 2 (receiving HIV-DNA with ZetaJet and EP) and group 3 (receiving HIV-DNA with ZetaJet and EP). An Env-specific reactivity was only seen in 1/22 (4%) vaccinees (S1 Table). This vaccinee received HIV-DNA without EP (group 1). No statistically significant difference was seen in the IFN-γ ELISpot response rate to Gag between HIV-DNA EP recipients (5/15, 33%) and vaccinees receiving HIV-DNA without EP (1/7, 14%, p = 0.6158).

**Fig 2:**
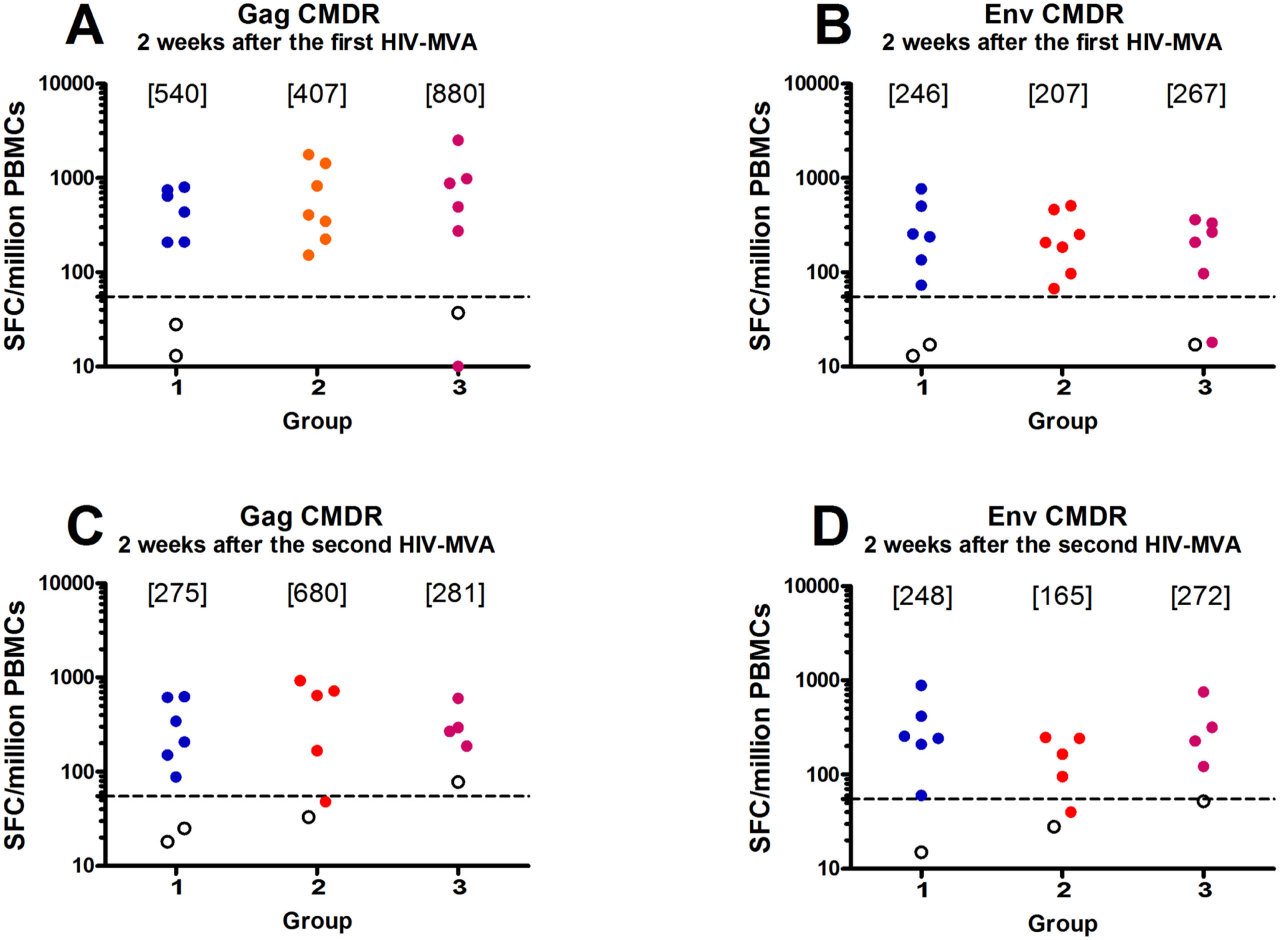
HIV-specific IFN-γ ELISpot responses. HIV-specific IFN-γ ELISpot responses two weeks after the first (upper panels) and second (lower panels) HIV-MVA/gp140/placebo vaccination to A) and C) Gag CMDR peptide pool stimulation and B) and D) Env CMDR peptide pool stimulation by HIV-DNA immunization groups. Cut-off was defined as >55 SFC/10^6^PBMCs and 4 times the background. The dashed line is set at 55. Placebo recipients are given as open circles. No statistically significant differences were found between groups.

The first HIV-MVA vaccination increased the overall response rate to 18/19 (95%), with 6/6 (100%), 7/7 (100%) and 5/6 (83%) responders to Gag or Env in groups 1, 2 and 3, respectively (Table 4). The overall magnitude in the 18 IFN-γ ELISpot responders was a median of 570 SFC/10^6^ PBMCs (range 152–2517) to Gag CMDR and a median of 245 SFC/10^6^ PBMCs (range 67–770) to Env CMDR. A majority of responders 12/18 (67%) exhibited broad IFN-γ ELISpot reactivity as seen by response to ≥ 3 HIV-DNA-specific peptide pools (S2 Table). The magnitude of IFN-γ ELISpot responses in responders to Gag CMDR was comparable across the groups, medians of 540 SFC/10^6^ PBMCs (range 208–802) in group 1, 407 SFC/10^6^ PBMCs (range 152–1778) in group 2 and 880 SFC/10^6^ PBMCs (range 275–2517) in group 3 (Fig 2a). The magnitude to Env CMDR in responders was also similar, with medians of 246 SFC/10^6^ PBMCs (range 73–770) in group 1, 207 SFC/10^6^ PBMCs (range 69–508) in group 2 and 267 SFC/10^6^ PBMCs (97–363) in group 3 (Fig 2b).

**Table 4:** IFN-γ ELISpot response rates in the three vaccinations groups after the first and second HIV-MVA boost.

**Peptide pool**	**Two weeks after 1** ^**st**^ **HIV-MVA boost, n = 19**			**Two weeks after 2** ^**nd**^ **HIV-MVA boost, n = 15**		
	Immunization group (no, %^a^)			Immunization group (no, %^a^)		
	Group 1	Group 2	Group 3	Group 1	Group 2	Group 3
****Gag CMDR****	6/6 (100%)	7/7 (100%)	5/6 (83%)	6/6 (100%)	4/5 (80%)	4/4 (100%)
****Env CMDR****	6/6 (100%)	7/7 (100%)	5/6 (83%)	6/6 (100%)	4/5 (80%)	4/4 (100%)
****Pol CMDR****	1/6 (17%)	2/7 (29%)	1/6 (17%)	0/6	0/5	0/4
****Gag or Env****	6/6 (100%)	7/7 (100%)	5/6 (83%)	6/6 (100%)	4/5 (80%)	4/4 (100%)

^a^Frequency of responders given as percentage of total number of evaluable vaccinees.

After the second HIV-MVA vaccination, the overall response rate was 14/15 (93%), with 6/6 (100%), 4/5 (80%) and 4/4 (100%) responders to Gag or Env in groups 1, 2 and 3, respectively (Table 4). The median responses to Gag CMDR in responders was 275 SFC/10^6^ PBMCs (range 88–627) in group 1, 680 SFC/10^6^ PBMCs (range 167–925) in group 2 and 281 SFC/10^6^ PBMCs (range 187–602) in group 3 (Fig 2c). The median response to Env CMDR in responders was 248 SFC/10^6^ PBMCs (range 60–883) in group 1, 165 SFC/10^6^ PBMCs (range 95–248) in group 2 and 272 SFC/10^6^ PBMCs (range 122–753) in group 3 (Fig 2d). The magnitude in responders to Gag CMDR peptide pool stimulation was statistically higher after the first HIV-MVA vaccination (a median of 696 SFC/10^6^ PBMCs) than after the second (a median of 319 SFC/10^6^ PBMCs, p = 0.0143). No difference was noted in the magnitude to Env CMDR peptide pool stimulation (median 246 SFC/10^6^ PBMCs versus 242 SFC/10^6^ PBMCs, p = 0.520, data not shown) between the first and second HIV-MVA boost.

#### ICS responses

HIV-specific CD4^+^ and CD8^+^ T cell responses to Gag and Env were determined four weeks after the first HIV-MVA boost. Of 18 vaccinees tested, 17 (94%) had CD4^+^ and/or CD8^+^ T cell responses to Gag and/or Env peptide pools, 14 (78%) in the CD4^+^ T cell compartment and 10 (71%) in the CD8^+^ T cell compartment. The CD4^+^ and CD8^+^ T cell responses to Gag and Env peptide pool stimulation are shown in S1 Fig.

**Fig 3:**
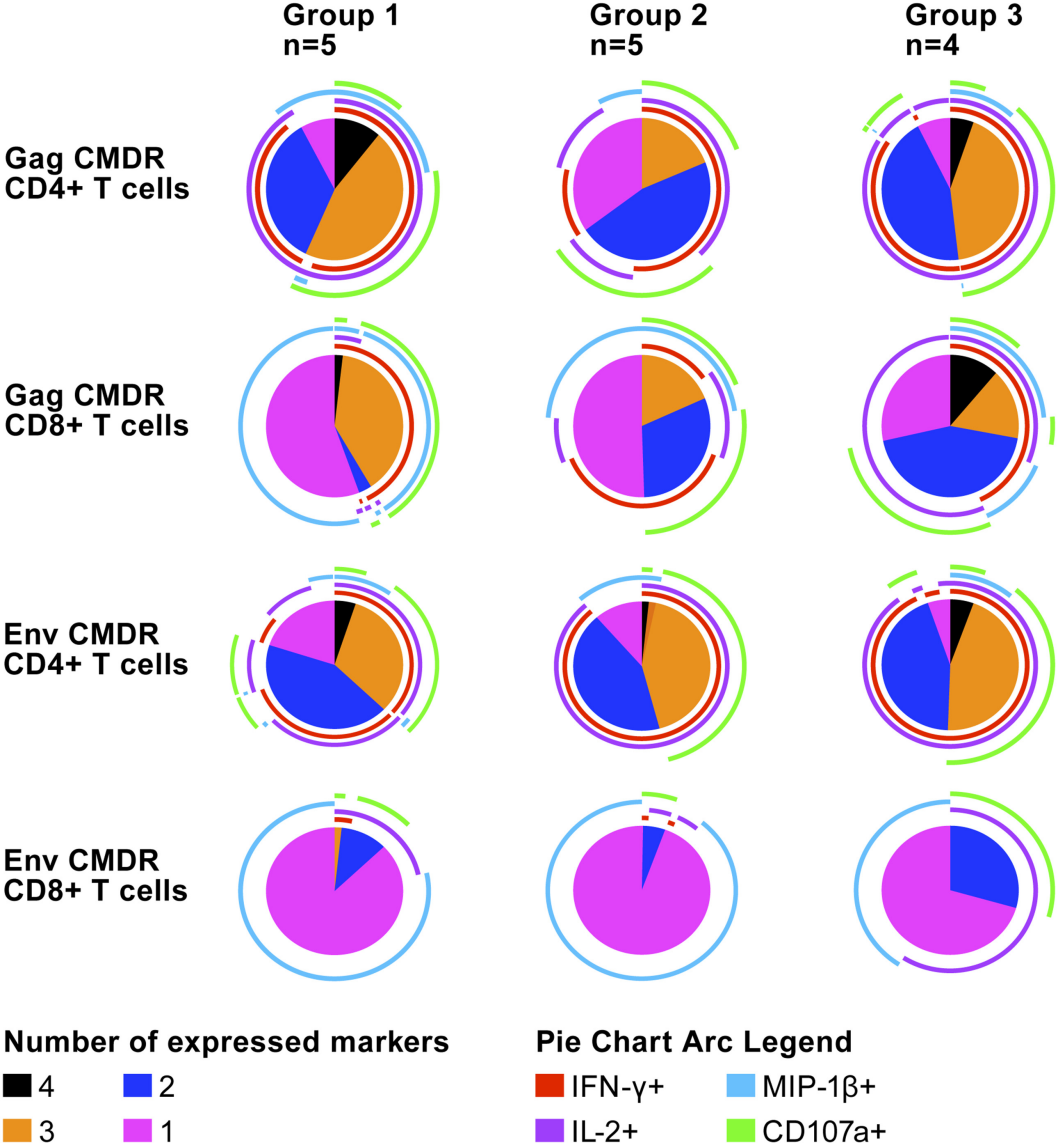
Polyfunctional analysis of Gag CMDR- and Env CMDR-specific responses of CD4^+^ and CD8^+^ T cells determined four weeks after the first HIV-MVA vaccination. The pie charts show the fraction of the responses based on the number of functions, one to four. The pie arcs show the relative contribution of each individual marker.

Polyfunctional analysis based on Boolean gating to create combinational events of IFN-γ, IL-2, MIP-1β and/or CD107, shown in Fig 3, revealed that overall approximately 45% of the Gag-responding CD4^+^ T cells had two functions, with predominance of IFN-γ/IL-2, and 40% were polyfunctional, predominantly expressing IFN-γ/IL-2/MIP-1β/CD107 or IFN-γ/IL-2/CD107. Among the Gag responding CD8^+^ T cells, approximately 25% had two functions, mainly producing IFN-γ/CD107 or IL-2/CD107 and 40% were polyfunctional, predominantly expressing IFN-γ/MIP-1β/CD107, IFN-γ/IL-2/MIP-1β or IFN-γ/IL-2/MIP-1β/CD107. Among Env- responding CD4^+^ T cells, approximately 45% had two functions, with predominance of IFN-γ/IL-2 expression, and 45% were polyfunctional, producing IFN-γ/IL-2/CD107, with some cells co-expressing MIP-1β. Most of the Env- responding CD8^+^ T cells (75%) were monofunctional, mainly expressing MIP-1β. The dual-functional cells (10%) expressed IL-2/CD107. No differences were seen between the groups.

#### Lymphocyte proliferation

**Fig 4:**
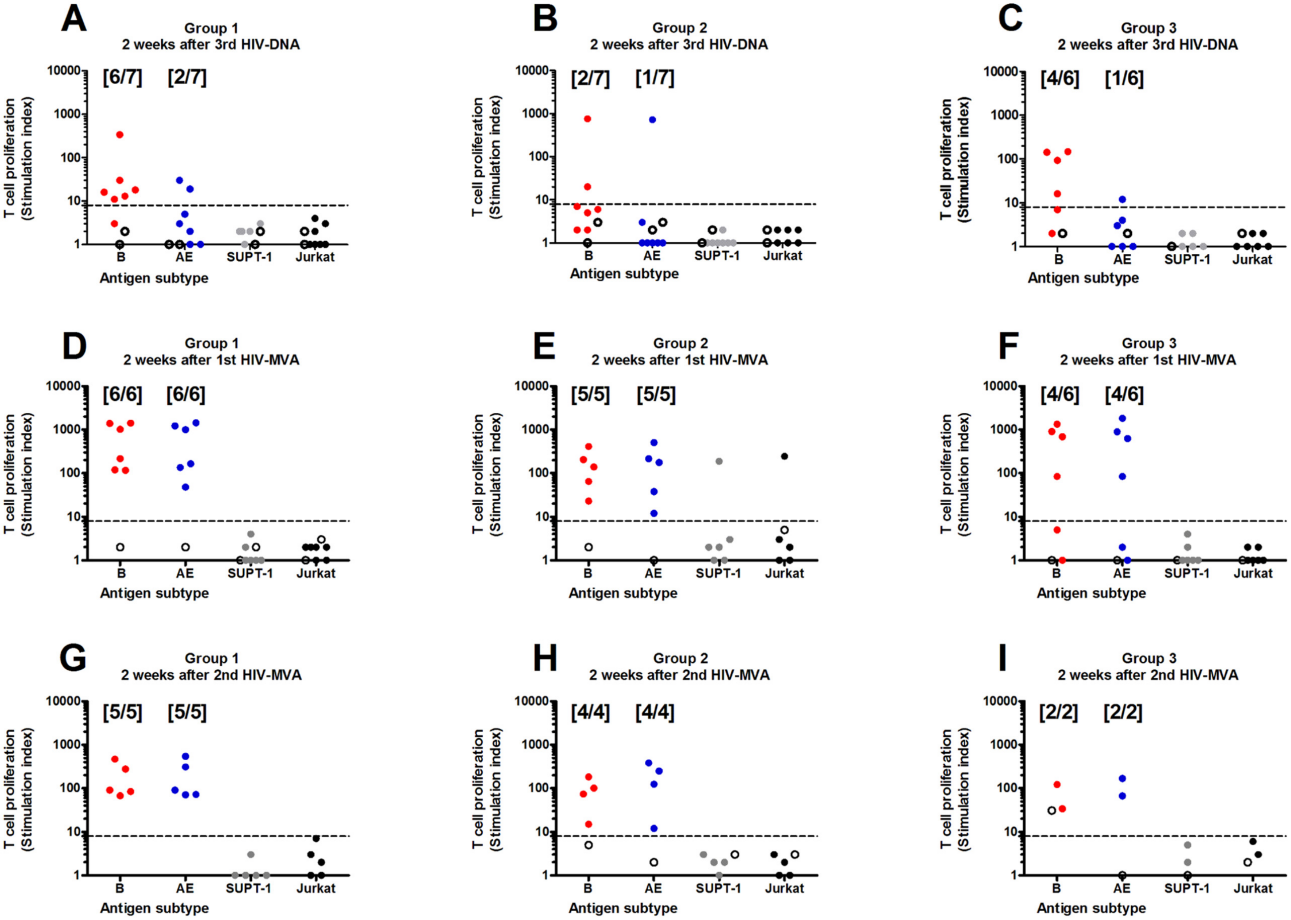
Lymphoproliferative responses. T cell proliferation against AT-2-inactivated-HIV-1 antigen as measured by [^3^H]-thymidine uptake assay two weeks after three HIV-DNA/placebo by vaccination groups (panels A-C), two weeks after the first HIV-MVA/gp140/placebo vaccination by vaccination groups (panels D-F) and two weeks after the second HIV-MVA/gp140/placebo vaccination by vaccination groups (panels G-I). Cut-off was set at SI ≥ 8 (dashed line). Placebo recipients are given as open circles.

T cell proliferation against AT-2-inactivated-HIV-1 MN and CM235 CRF01_AE antigen was measured by [^3^H]-thymidine uptake assay two weeks after three HIV-DNA/placebo immunizations and two weeks after the first and second HIV-MVA/gp140/placebo vaccination. The T cell proliferative responses are shown in Fig 4. Two weeks after receipt of three HIV-DNA immunizations the overall frequency of responders was significantly higher to MN subtype B (12/20, 60%) than to the CM235 CRF01_AE antigen (4/20, 25%; p = 0.0225). Among the vaccinees who received the HIV-DNA vaccine without EP, 6/7 (85%) responded in the T cell proliferation assay to MN subtype B stimulation. Although not significant (p = 0.1577), fewer vaccinees receiving HIV-DNA in combination with EP exhibited MN subtype B-induced T cell proliferative responses (6/13, 46%) than those without EP.

The first HIV-MVA/gp140 vaccination increased the T cell proliferative response rate to 15/17 (88%) to both MN subtype B (p = 0.0726) and CM235 CRF01_AE antigen (p<0.0001). After this first HIV-MVA/gp140 vaccination high stimulation indices to MN subtype B were noted in all groups, with medians of SI 619 (range 117–1421) in group 1 SI 103 (23–411) in group 2 and SI 799 (84–1348) in group 3. Similarly, high stimulation indices were noted in response to CM235 CRF01_AE, with a median of SI 583 (range 48–1436) in group 1, SI 102 (range 12–506) in group 2 and SI 761 (range 84–1833) in group 3 (Fig 4).

Following the second HIV-MVA/gp140 vaccination the response rate to both MN subtype B and CM235 CRF01_AE antigen was 11/11 (100%). T cell proliferative responses were similar after the first and second HIV-MVA vaccinations. In vaccinees where data was available at both time points (n = 9), responses to MN subtype B were SI 206 (range 21–1421) after the first HIV-MVA vaccination and SI 91 (range 21–469) after the second HIV-MVA (p = 0.1289) and responses to CM235 CRF01_AE were SI 216 (range 12–1436) after the first HIV-MVA and SI 124 (range 27–544) after the second HIV-MVA (p = 0.2500).

### Antibody-mediated immune responses

**Fig 5:**
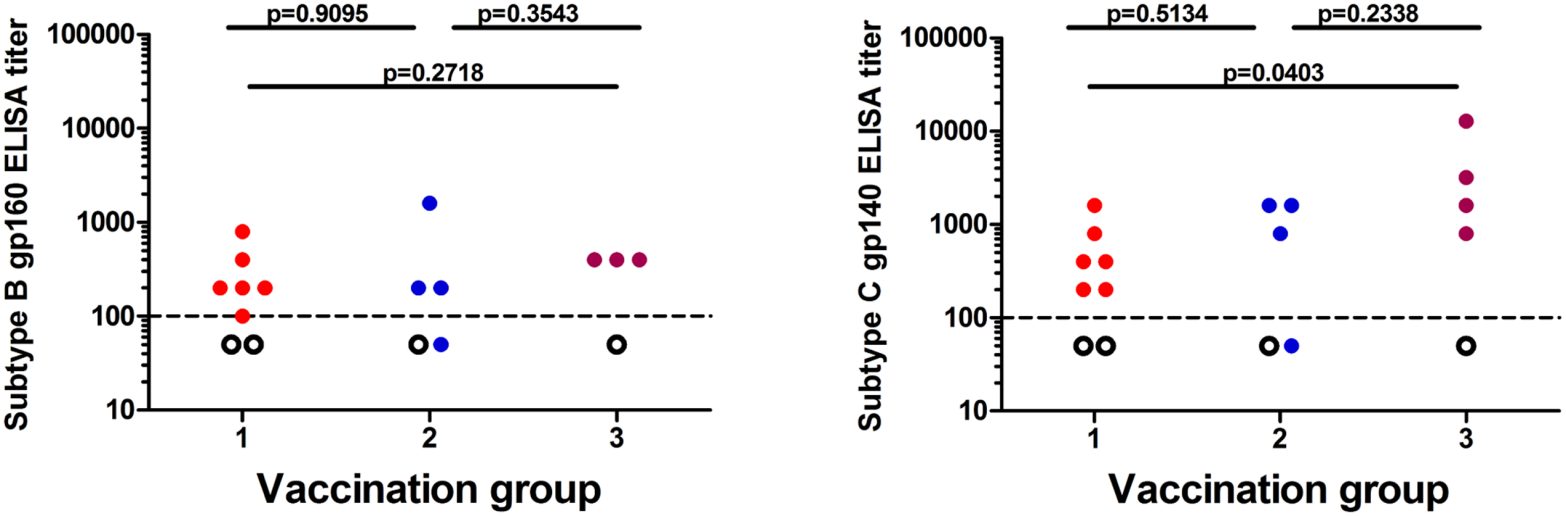
Binding antibody responses of vaccinees tested four weeks after the second HIV-MVA vaccination. Binding antibody titers to subtype B (IIIB) gp160 (A) and to subtype C (HIV-1 96ZM651) gp140 (B) envelope proteins are shown. The lowest dilution used was 1:100. The dashed line is therefore set at 100. Negative values were arbitrarily given a value of 50. Placebo recipients are given as open circles.

None of the vaccinees, irrespective of vaccination group, demonstrated anti-gp160 antibodies after the first HIV-MVA vaccination. Four weeks after the second HIV-MVA vaccination binding antibodies were demonstrated in almost all of the vaccinees to subtype B gp160 (11/13, 92%) and subtype C gp140 (13/14, 93%), while all four placebo recipients were negative for both proteins. The overall antibody ELISA titer to the subtype B gp160 was 200 (range <100–1600), while that to the subtype C gp140 was 800 (range <100–12800; Fig 5). No difference in median anti-subtype B gp160 antibody titer was seen between the three vaccination groups. The median anti-subtype C gp140 antibody titer was 400 (range 200–1600) in group 1, 1200 (<100–1600) in group 2 and 2400 (800–12800) in group 3. Vaccinees in group 3 (receiving DNA priming with EP plus HIV-MVA and subtype C gp140) had a statistically higher median titer than those in group 1 (receiving DNA priming without EP plus HIV-MVA without subtype C gp140), p = 0.0403 (Fig 5).

Neutralizing antibody activity was determined in the 13 samples exhibiting anti-Env antibodies four weeks after the second HIV-MVA vaccination. There was no demonstrable neutralizing antibody activity using the TZM-bl pseudovirus assay and either subtype B SF162 or subtype C MW965.26 PSVs, nor were any neutralizing antibodies detected using a PBMC-based assay with either subtype B SF162 or CRF01_AE CM244 virus (data not shown). Thus, despite a high frequency of binding activity to envelope the antibodies did not show a neutralizing activity to Tier 1 viruses of subtypes B and C or Tier 2 CRF01_AE.

## Discussion

In the present hypothesis-generating clinical HIV vaccine trial we explored the safety, tolerability and immunogenicity of three intradermal HIV-DNA priming immunizations with or without intradermal EP followed by boosting twice with HIV-MVA given intramuscularly. A group of vaccinees were randomized to receive CN54 subtype C gp140 protein (without adjuvant) at the time of the HIV-MVA vaccinations. Thirty healthy volunteers were enrolled in the trial, of whom 19 volunteers (including three placebo recipients) received EP. The vaccinations were safe and very well tolerated.

Intradermal immunization with the Zetajet was well tolerated and addition of ID EP with the current device and settings added little discomfort. No volunteer hesitated to complete the study schedule. In a small series an increased electroporated ID volume, 3 mm needles with a gap of 6 mm with a corresponding increase in voltage, was feasible (Hejdeman and Sandström, personal observation). This opens for further studies of needle-free administration of vaccines and evaluation of ID EP.

This is the first clinical report of intradermal EP used in combination with ID HIV-DNA (0.6 mg) vaccinations delivered using ZetaJet. After three HIV-DNA immunizations, IFN-γ ELISpot response rates to Gag peptide pool stimulation were modest and not statistically different between vaccinees who received HIV-DNA with EP (5/15, 33%) compared to those receiving HIV-DNA without EP (1/7, 14%). HIV-Env-specific binding antibodies were not detected after the first HIV-MVA/gp140 vaccination, but after the second HIV-MVA/gp140 vaccination. Previous clinical HIV vaccine trials using EP have applied intramuscular delivery of the DNA vaccine in combination with intramuscular EP. Vasan et al reported a 63% ELISpot response rate in vaccinees receiving 1.0 mg ADVAX DNA and intramuscular EP (TriGrid delivery System) after two vaccinations, while none of the eight vaccinees receiving a higher ADVAX dose (4.0 mg) without EP developed ELISpot reactivity [29]. The use of 3 mg PENNVAX-B DNA vaccine in combination with intramuscular EP with the CELLECTRA Adaptive Constant Current Electroporation device (Inovio Pharmaceuticals) induced HIV-specific CD4^+^ and CD8^+^ T cell responses to Gag and/or Env in 3/10 (30%) vaccinees after two vaccinations and in 4/9 (44%) after three vaccinations [30]. Binding antibody responses to Env were rare in both trials [29,30].

We have performed a small pilot study where a single vaccination of 10^8^ pfu HIV-MVA was administered IM to ten healthy Swedish vaccinees. Two weeks after the vaccination, 3 (33%) of 9 evaluable vaccinees had a Gag-specific IFN-γ ELISpot response. The magnitude of response in the three responders was 85, 130 and 75 SFC/10^6^ PBMCs, respectively. The same vaccinees also had Env-specific responses of 95, 75, and 60 SFC/10^6^ PBMCs, respectively (Biberfeld, Earl, Hejdeman, Nilsson, Robb, Sandström, unpublished data). Here, after receipt of three HIV-DNA immunizations, the first HIV-MVA vaccination increased the overall IFN-γ ELISpot response rate to 18/19 (95%) and response rates were similar across the vaccination groups. All IFN-γ ELISpot responders exhibited broad reactivity, as seen by responses to multiple peptide pools and the magnitude of responses was high to both Gag and Env peptide pools (medians of 570 and 245 SFC/10^6^ PBMCs, respectively). These findings are similar to those described in the HIVIS03 trial, where vaccinees received three HIV-DNA vaccinations of 1 mg ID or 3.8 mg IM delivered using a needle-free device (Biojector) followed by HIV-MVA. In that trial, using freshly prepared PBMCs for testing, an overall IFN-γ ELISpot response rate of 100% was observed with a response to multiple peptide pools and median responses to Gag and Env of 625 and 267 SFC/10^6^ PBMCs, respectively [21].

In the present trial, HIV-specific CD4^+^ and CD8^+^ T cell responses to Gag or Env peptides were seen in 17/18 (94%) vaccinees tested by ICS after the first HIV-MVA vaccination. A balanced response was seen with 78% responding in the CD4^+^ compartment and 71% in the CD8^+^ compartment. IFN-γ and IL-2 dominated the CD4^+^ T cells responses to Gag and Env. A high degree of polyfunctionality, with co-expression of MIP-1β and/or the degranulation marker CD107 was also noted. The CD8^+^ response to Gag was dominated by IFN-γ and MIP-1β, combined with IL-2 and/or CD107. CD8^+^ T cell reactivity to Env was rare and induced monofunctional cells mainly expressing MIP-1β. These findings are similar to those we reported previously when using the same vaccines, but with a delayed second HIV-MVA boost [39]. Notably, no clear differences were seen between the vaccination groups. In the trial studying ADVAX DNA and intramuscular EP (TriGrid delivery System), EP delivery improved the quality of the T-cell response by inducing parallel secretion of IFN-γ, IL-2, TNFα and MIP-1β [29].

Binding antibodies to subtype B gp160 and subtype C gp140 were detected in a high proportion (92% and 93%, respectively) of vaccinees’ sera four weeks after the second HIV-MVA vaccination. No difference in median titer to gp160 was seen, whether or not EP was used for priming. Notably, the vaccinees who received three HIV-DNA vaccinations in combination with intradermal EP prior to the two HIV-MVA vaccinations combined with gp140 protein boost (group 3) exhibited significantly higher gp140 binding ELISA titers than volunteers given HIV-DNA without EP and HIV-MVA vaccinations without gp140 (group 1, p = 0.0403). Nonetheless, the titers in HIV-DNA/EP plus HIV-MVA/gp140 recipients (group 3) were not significantly higher than in vaccinees who received HIV-DNA/EP and HIV-MVA alone (group 2), suggesting minimal influence of gp140 on boosting for binding antibodies.

Here we administered the Envelope protein without an adjuvant since a suitable adjuvant was not available and the purpose of including the gp140 protein boost in the present trial was primarily to assess the safety of the protein in preparation for a larger clinical trial. The Env gp140 subtype C boosts given in combination with HIV-MVA vaccinations did not notably enhance the Env-specific cellular immune responses as determined by ELISpot, LPA and ICS. Further studies are needed to determine whether administration of the protein given with an appropriate adjuvant will enhance antibody responses.

The limitation of this trial was the low number of participants, partly due to the low remuneration and the high visit requirements of the trial, which also caused some loss to follow-up. However, the number of electroporations that were given indicates the tolerability of this intervention. The lack of convincing effect of ID EP observed here has to be verified in a larger study.

We have thus demonstrated that ID EP is safe and highly tolerable in healthy volunteers. Strong cell-mediated and antibody-mediated immune responses were elicited by the HIV-DNA prime HIV-MVA boosting regimen, irrespective of ID EP use. A phase IIa trial where ID HIV-DNA delivery is administered using the ZetaJet device with or without EP and adjuvanted gp140 subtype C is given simultaneously with HIV-MVA vaccinations is ongoing in two African countries.

## Supporting Information

S1 CONSORT Checklist CONSORT checklist. (DOCX)

S1 Fig CD4^+^ and CD8^+^ T cell responses to Gag CMDR and Env CMDR peptide pool stimulation four weeks after the first HIV-MVA vaccination. Eight-colour ICS was performed on cryopreserved PBMCs and expression of IFN-γ, IL-2, MIP-1β and CD107a was assessed. CD4^+^ T cell responses to A) Gag, and B) Env as well as CD8^+^ T cell responses to C) Gag and D) Env are shown.
(TIF)

S1 Protocol HIVIS07 study protocol. (DOC)

S1 Table IFN-γ ELISpot response rates after three HIV-DNA immunizations. (DOCX)

S2 Table IFN-γ ELISpot response rates to peptide pools representing the HIV-DNA sequence. (DOCX)
