# Supplementary figures and images for: HIV-DNA Given with or without Intradermal Electroporation Is Safe and Highly Immunogenic in Healthy Swedish HIV-1 DNA/MVA Vaccinees: A Phase I Randomized Trial
Source: PLoS One. 2015 Jun 29;10(6):e0131748. doi: 10.1371/journal.pone.0131748 (PMC4486388; doi:10.1371/journal.pone.0131748)

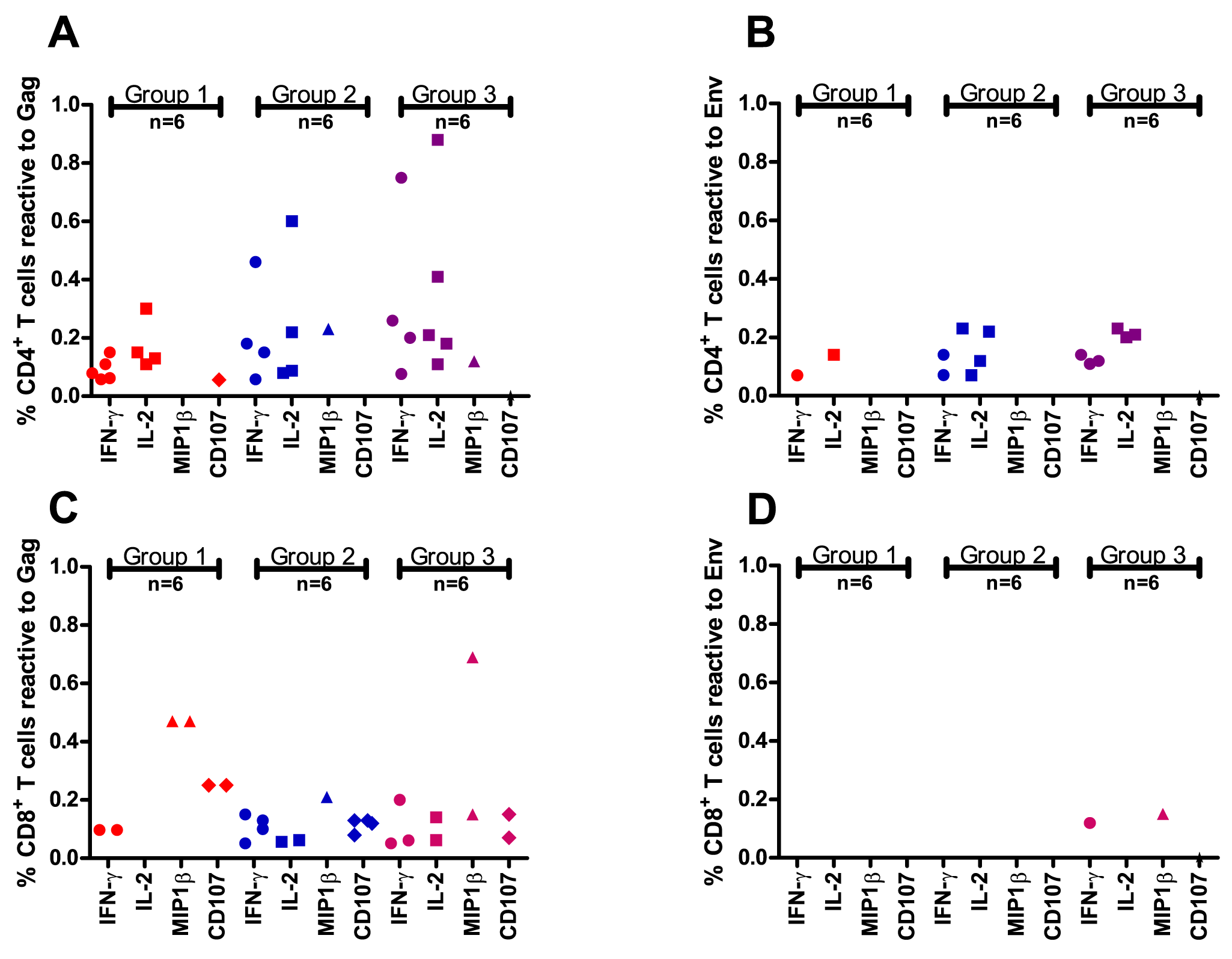

Supplement: S1 Fig — Eight-colour ICS was performed on cryopreserved PBMCs and expression of IFN-γ, IL-2, MIP-1β and CD107a was assessed. CD4+ T cell responses to A) Gag, and B) Env as well as CD8+ T cell responses to C) Gag and D) Env are shown. (TIF) [file pone.0131748.s002.tif]
