# Supplementary material for: HIV-DNA Given with or without Intradermal Electroporation Is Safe and Highly Immunogenic in Healthy Swedish HIV-1 DNA/MVA Vaccinees: A Phase I Randomized Trial
Source: PLoS One. 2015 Jun 29;10(6):e0131748. doi: 10.1371/journal.pone.0131748 (PMC4486388; doi:10.1371/journal.pone.0131748)
Supplement: S1 Protocol — (DOC) [file pone.0131748.s003.doc]

Modifications of version 1.4 approved by the Ethics committee

# HIVIS 07

# A phase I trial to assess the safety and feasibility of administering plasmid DNA carrying multiple HIV-1 genes together with dermal electroporation

(HIVIS 07. En fas I studie för att studera säkerheten och användbarheten av att immunisera med plasmid DNA med infogade HIV gener med dermal electroporering)

The trial is funded through Sida/SAREC

Financial or intellectual support has also been received by AVIP, Europrise, VR, SMI/KI, Vecura, CytoPulse, Bioject and Walter Reed Army Institute for Research for the design, preclinical trials and research to establish the vaccines.

**Sponsor**

The Swedish Institute for Infectious Disease Control

For the sponsor ...................................................................... Date ...................................

Johan Carlson

Director General, SMI

For the protocol team................................................................Date..................................

Bo Hejdeman

# The trial will be conducted at South Stockholm General Hospital (Södersjukhuset)

Principal Investigator Bo Hejdeman Bo.Hejdeman@sodersjukhuset.se

Department of Venhälsan

Karolinska Institutet

Södersjukhuset AB

118 83 Stockholm, SWEDEN Fax: 00 46 8 616 2509

Clinical Investigator Eric Sandström Eric.Sandstrom@sodersjukhuset.se

Department of Venhälsan

Karolinska Institutet

Södersjukhuset AB

118 83 Stockholm, SWEDEN Fax: 00 46 8 616 2509

# The trial will be executed at Dept Venhälsan, South Stockholm General Hospital (Södersjukhuset)

Trial nurses Inger Petz

Stefan Ekroth

PhD Lars Eriksson

Dept Venhälsan

South Stockholm General Hospital

118 83 Stockholm, SWEDEN Fax:00 46 8 616 2509

Monitor Birgitta Strandberg

Karolinska Trial Alliance (KTA)

Hälsingegatan 43

171 76 Stockholm

**DNA plasmids will be supplied by**

Professor Britta Wahren Britta.Wahren@smi.se 00 46 8 457 2630

Department of Virology

The Swedish Institute for Infectious Disease Control

# 171 82 Solna Tel 00 46 8 457 2630 Fax: 00 46 8 337272

# Electroporation device, DermaVax, supplied by CytoPulse

# CytoPulse Electroporation and Electrofusion System

# 810 Cromwell Park Dr. Suite T

# Glen Burnie, MD 21061, USA

# [**www.cytopulse.com**](http://www.cytopulse.com/)

# ZetaJet® will be supplied by

Bioject Medical Technologies, Inc.
20245 S.W. 95th Avenue
Tualatin, Oregon 97062
Toll Free: 800.683.7221
Phone: 503. 692.8001
Fax: 503.692.6698

# Laboratory Investigations will be conducted at the Swedish Institute for Infectious Disease Control

Professor Gunnel Biberfeld

Department of Immunology and vaccinology

gunnel.biberfeld@smi.se, Tel: 0046 8 4572660

Professor Britta Wahren

Department of Virology

britta.wahren@smi.se, Tel: 0046 8 4572630, Fax: 0046 8 337272

The Swedish Institute for Infectious Disease Control

171 82 Solna

Protocol team

Biberfeld, Gunnel,

Bråve, Andreas

Hejdeman, Bo; *Chair*

Marovich, Mary

Nilsson, Charlotta

Sandström, Eric; *Co-chair*

Wahren, Britta

Trial Co-ordinating Committee, TCC

Biberfeld, Gunnel,

Bråve, Andreas

Hejdeman, Bo; *Chair*

Marovich, Mary

Nilsson, Charlotta

Sandström, Eric; *Co-chair*

Wahren, Britta

Steering Committee, SC

Andersson, Sören

Biberfeld, Gunnel

Hejdeman, Bo

Norrby, Ragnar

Sandström, Eric

Wahren, Britta

Trial Management Group, TMG

Ekroth Stefan

Hejdeman Bo

Petz Inger

Sandström Eric

Laboratory Management Group, LMG

Andersson, Sören

Biberfeld, Gunnel

Broliden, Kristina

Bråve, Andreas

Nilsson, Charlotta

Wahren, Britta

Data and Safety Monitoring Committee, DSMC

Peter Liljeström Chair

Patrick Olin Clinician

Anders Sönnerborg Clinician

HIVIS 07 team

Andersson, Sören

Biberfeld, Gunnel

Broliden, Kristina

Bråve, Andreas

Hejdeman, Bo

Nilsson, Charlotta

Sandström, Eric

Wahren, Britta

**Contents**

Synopsis

Introduction

1. General plan
   1. Objectives
   2. Design
   3. Population
   4. Trial products
   5. Endpoints
2. Schedule of visits
   1. Recruitment
   2. Screening
   3. Trial entry
   4. Randomisation and study procedures
   5. Final visit (including in event of a discontinuation)
   6. Procedural Flow Chart
3. Procedures
   1. Blood and urine collection
   2. Clinical history and examination
   3. Genital infection screen
   4. Pre-HIV test screen and counselling
   5. Safer sex counselling and condom provision
   6. HIV related issues (see also section 6)
   7. Discontinuation procedures (including withdrawal and stopping the study)
   8. Unblinding procedure
4. Assessments
   1. Safety assessments
   2. Immunogenicity assessments
   3. Other assessments
5. Adverse events
   1. Definitions
   2. Relationship to study product
   3. Reporting adverse events
   4. Clinical management
6. Management of HIV issues during and following the trial
   1. HIV testing
   2. HIV infection
   3. Social discrimination as a result of a post-vaccine response
7. Management of the trial
   1. Data management at the clinical centres
   2. Data management in the immunology laboratories
   3. Data management
   4. Monitoring
   5. Data ownership
   6. Trial Committees (SC and TMG)
   7. HIVIS Steering Committee
   8. Data and Safety Monitoring Committee (DSMC)
8. Statistical considerations
   1. Sample size
   2. Analysis

9 Confidentiality, ethics and responsibilities including indemnity

10 Protocol amendments

11. Publication

12 References

*Appendices*

Clinical Report Forms

**Synopsis**

# TITLE: A phase I trial to assess the safety and feasibility of administering plasmid DNA carrying multiple HIV-1 genes together with dermal electroporation

**Objectives**

*Primary*

Is it safe and tolerable to immunize with seven or eleven DNA plasmids carrying HIV-1 genes with electroporation?

*Secondary*

1. Is it possible to increase immunogenicity of a DNA vaccine delivered intradermally and to reduce the number of injections needed by using electroporation?
2. Will electroporation improve/alter the quality of the immune responses induced by the HIV-1 DNA plasmids?
3. Is it possible to obtain similar immune reactivity by replacing two intramuscular boosts using MVA with two intradermal injections of HIV-1 DNA plasmids together with electroporation?
4. Can electroporation increase immunogenicity of all included DNA plasmids to permit mixing of all vaccine plasmids instead of delivering them as two different entities?
5. Will additional DNA plasmids broaden the anti-HIV-1 immune response?

**Study participants**

The study population consists of 48 healthy adults who are not infected by HIV. Subjects will be men or women, 18 to 40 years of age. Volunteers must not be engaging in high-risk behaviour for HIV (i.e., drug injection or sex with HIV positive partner). Participants will agree to practice safer sex to avoid infection with sexually transmitted infections and to use contraceptives to prevent pregnancy. For men this will mean consistent condom use and encouragement for partners to use oral contraception or IUD and for women mandatory oral contraception, IUD use or equivalent contraception in addition to consistent condom use.

Number of volunteers: 48 volunteers

Clinical site: South Stockholm General Hospital, Stockholm, SWEDEN

Clinical laboratories: Karolinska University Hospital, Huddinge, SWEDEN

Immunology and virology

laboratories: The Swedish Institute for Infectious Disease Control, Stockholm,

SWEDEN

*Study design*

| **Group** | **N** | **Mode of Immunization**  **(w 0, 6 , 12)** | **Mode of delivery** | **Left arm (Ampoules 1**  **env)** | | **Right arm (Ampoules 2**  **gag, Rtmut)** | **Boost immunization**  **(w 36 and 60)** |
| --- | --- | --- | --- | --- | --- | --- | --- |
| B1 | 10 | Id  ZetaJet® | No electroporation | 1 inj 100µl  0,3 mg DNA* | 1 inj100µl  0,3 mg DNA* | | 1 im inj needle  MVA 10^8^ pfu |
|  | 2 | Id  ZetaJet® | No electroporation | 1 inj 100µl saline | 1 inj 100µl saline | | 1 im inj needle  100µl saline |
| B2 | 10 | Id  ZetaJet® | electroporation | 1 inj 100µl  0,3 mg DNA* | 1 inj 100µl  0,3 mg DNA* | | 1 im inj needle  MVA 10^8^ pfu |
|  | 2 | Id  ZetaJet® | electroporation | 1 inj 100µl saline | 1 inj 100µl saline | | 1 im inj needle  100µl saline |
| B3 | 10 | Id  ZetaJet® | electroporation | 1 inj 100µl  0,3 mg DNA* | 1 inj 100µl  0,3 mg DNA* | | 2 id inj Zetajet®,  tot. 0,6 mg DNA + electroporation |
|  | 2 | Id  ZetaJet® | electroporation | 1 inj100µl saline | 1 inj100µl saline | | 2 id inj Zetajet®,  tot 200µl saline +  electroporation |
| B4 | 10 | Id  ZetaJet® | electroporation | 1 inj100µl  0,3 mg DNA** mixed ampoules | 1 inj100µl  0,3 mg DNA** mixed ampoules | | 1 im inj needle  MVA 10^8^ pfu |
|  | 2 | Id  ZetaJet® | electroporation | 1 inj100µl saline | 1 inj100µl saline | | 1 im inj needle  100µl saline |

*) 7 DNA plasmids **) 11 DNA plasmids.

Immunizations will be given as prime injections (HIV DNA) at weeks 0, 6 and 12 and as boost injections (MVA) at week 36 and week 60.

Groups B1, B2 and B3 will be randomised together. Laboratory will be blinded as to groups B1, B2 and B3. Volunteers will be recruited separately to group B4. Both the clinic and the laboratory will be blinded as to controls in each separate group.

*Duration of study*

The enrolment period is expected to be 12 months. Immunizations will be made over 84 weeks. Subjects will be monitored for 12 weeks after the last vaccine dose. The total study period after enrolment is 72 weeks.

*Description of candidate vaccines*

The seven DNA plasmids that are the compounds in the vaccine that will be used in groups B1, B2 and B3 contain~~s~~ the following sequences: envelope (env) of HIV-1 subtypes A, B and C; gag of subtypes A and B; reverse transcriptase and rev; both of subtype B. This plasmid DNA composition has been used in two phase 1 studies in Sweden and Tanzania (Sandström et al., 2008; Bakari et al., 2008, 2009). The eleven DNA plasmids which are the compounds in the vaccine that will be used in group B4 (all plasmids mixed) in addition contains gag subtype C and nef, tat and viral protease, the three latter of subtype B. These additional constructs are motivated since subtype C is a prevalent infection in Africa and the gag C compound will enhance the broad cellular immune response. The conserved nef and tat early genes have previously been used by us in human studies (Calarota et al., 1998 and 1999) and induce cellular immunity to the respective antigens in humans. The viral protease gene is included to enhance responses to the viral enzymes, since the RT gene has a low immunogenicity in vaccination although it becomes a strong CTL-inducing gene in late HIV infection. For this reason we will retain the RT gene in the plasmid composition.

The MVA-CMDR contains the env genes from subtype E and the gag and pol genes of subtype A. The gag and pol genes of subtype A are homologous, in terms of subtype to the gag A sequence in the DNA plasmids used for the priming immunizations, although not identical in sequence. The env genes are less related to the sequences in the DNA plasmids but have been shown to broaden and strengthen the plasmid DNA primed anti-env immune responses.

*Product administration*

DNA: The plasmids are produced by Vecura, Huddinge, Stockholm, SWEDEN for The Swedish Institute for Infectious Disease Control, Stockholm, SWEDEN. This vaccine contains no live microbial vector.

MVA: The HIV MVA-CDMR is produced by the US Military HIV Research Program, Rockville, Maryland, USA. This vaccine contains the live attenuated Modified vaccinia virus Ankara with recombinant HIV-1 genes.

ZetaJet® is a jet stream delivery device produced by Bioject Medical Devices that is used to administer DNA plasmids in skin or muscle. The ZetaJet® is used for the id administration of the HIVIS DNA just before electroporation. Contact; Richard Stout, Bioject Medical Technologies, Inc., 20245 S.W. 95th Avenue, Tualatin, Oregon 97062. Toll Free: 800.683.7221

*Description of electroporation*

We are proposing to use a device, DermaVax™ (Cyto Pulse Sciences, Inc.) adapted for dermal electroporation in conjunction with intradermal injections of plasmid DNA. This technique uses microelectrodes to prevent skin damage and reduce the discomfort that is associated with intramuscular electroporation. The use of intradermal injection + electroporation, in contrast to intramuscular treatment, also allows for direct ocular inspection of the treatment sites.

DermaVax™ is produced by Cyto Pulse Sciences, Inc, 810 Cromwell Park Drive, Suite T, Glen Burnie, MD 21061, USA; a company developing novel electric field based technologies for cancer treatment, gene therapy and infectious disease applications. The devise aids in introducing plasmids intracellularly for increased expression.

*Endpoints*

Primary endpoints:

1. The safety of immunization with the seven or eleven DNA plasmids carrying HIV-1 genes and concomitant electroporation will be assessed by clinical signs and standard biochemical and haematological laboratory tests. Any grade of severity will be considered for causality by the vaccine. A fourfold increase in two consecutive tests will be considered significant.
2. The alternative boost immunization (im with MVA or id with HIV DNA + electroporation) will be evaluated by assessing local (pain and cutaneous indurations), general (fever, chills, headache, nausea, vomiting, malaise, myalgia) and other unsolicited adverse events within 28 days. Any grade III or IV event will be taken as an indication that the treatment in the specific arm is less tolerable.

Secondary endpoints:

A qualitative evaluation of cellular and humoral immune responses against antigens representing the different immunogens will be performed.

**Introduction**

*General aims of the trial*

The HIV infection is one of the most lethal infectious diseases today. Around 30-40 million persons are infected, and may thus transmit the virus. A vaccine would be the most efficient way to stop transmission and in the long run eradicate disease (similar to what was achieved against smallpox). To vaccinate against HIV is difficult since a few particles may be enough to infect the host. The virus genome then remains incorporated in the infected cell and can replicate at any time after infection. The virus genome is variable and therefore immune responses to one subtype may not protect against virus of another subtype. Antibody responses are needed to neutralize the primary infectious virus and a cell mediated immunity is needed to eliminate the first infected host cells and, in the case of an infection, suppress the viral replication.

A vaccine based on the delivery of genetic material, either in the form of a plasmid or a viral vector, gives rise to a production of the vaccine antigen (protein) in the body of the vaccinee. A protein produced in this manner will be naturally processed (i.e. correctly folded and glycosylated) thus mimicking the proteins carried by the virus. To deliver the plasmid DNA by electroporation will permit a higher number of vaccine-plasmids to enter skin cells, which then start producing the vaccine antigen. These antigens are considered foreign to the host, and evoke both cellular and antibody immune responses. Therefore the proposed HIV vaccination protocol with electroporation should permit a lower dose of the HIV genes to be delivered and still induce a potent immune response. The mixture of genes from several subtypes of HIV will at the same time permit a broader immune response to several subtypes of HIV.

*Preliminary results*

A phase I trial (HIVIS 01/02) with a multi-subtype HIV-1 DNA vaccine boosted with a vaccinia vector (MVA) with the corresponding HIV-genes inserted into it, has been successfully conducted in our clinic in Sweden. The combination of DNA and MVA was proven safe and highly immunogenic as 34/37 (92%) of participants reached the primary endpoint of positive IFN gamma ELISPOT of whom 31 reacted to gag peptides and 24 to env peptide pools. Including also other assays of HIV-specific immune responses, 97% (all but one subject) of the cohort participants, developed HIV-specific immune responses. All DNA injections were given by the Biojector2000 needle-free device. Preliminary analysis indicates that ¼ the dose DNA given id primes an immune response equally as well as a ‘standard’ 4 mg dose given im, and that boosting with MVA could be achieved in the presence of vaccinia immunity. Volunteers above 40 years of age mounted a significantly lower immune response to the immunogens. It was also evident that the higher dose of MVA (10^8^ pfu) delivered im gave better boosting of the DNA primed responses than when 10^7^ pfu of MVA was given id. However, in order to administer the DNA intradermally, five shots with the Biojector were required at each immunisation time point. In contrast to the prominent T cell responses detected after immunization only weak anti-HIV antibody responses were observed (Sandström et al., 2008).

A further placebo-controlled phase II trial (HIVIS 03) in Dar es Salaam (MUHAS) of 60 non-infected individuals has confirmed the safety and immunogenicity of the HIVIS DNA and HIV MVA boost schedule. The study is now unblinded and analysis presented at the AIDS vaccine meeting in Paris 2009 (Bakari, et al., 2009). Since 1986 laboratory and clinical capacity building has taken place in Dar es Salaam, Tanzania, through the SIDA/Sarec TANSWED programme. This has led to the introduction of HIV vaccine studies in line with the Tanzanian National HIV Vaccine Strategic framework under the European Community program HIVIS, supported by both SIDA/Sarec and EU. An extension of this trial, TaMoVac has been funded by EDCTP. It is also anticipated that a further trial will be needed to introduce the technique for administration of vaccines (electroporation). This has passed through all the application steps for funding from EDCTP.

*HIV vaccine strategies under investigation*

There is a broad scientific consensus that a successful vaccine to prevent HIV-1 transmission must be able to elicit HIV-specific CD8+ cytotoxic T-lymphocytes (CTL) and also antibodies capable of neutralising primary HIV isolates (Nab). Present vaccine candidates against HIV-1 include: protein or peptide subunit vaccines with adjuvants; live-vector based vaccines; and plasmid DNA vaccines.

Subunit vaccines, such as highly purified recombinant monomeric HIV-1 envelope proteins elicit low or no virus-specific CTL, strongly binding but not broadly neutralizing antibody responses, even when adjuvanted with potent immunostimulants. Two HIV vaccine efficacy trials with HIV-1 subtypes B and E glycoproteins gp120 have been completed. The vaccine did not induce protection against HIV-1 infection, nor did it lower the viral load after infection. A phase III trial with the gp120 subunit vaccine combined with a canarypox vector encoding the same protein was shown to have a moderate protective effect of 31.2% (Rerks-Ngarm et al., 2009).

A phase IIB trial, termed STEP, of an adenovirus vector based vaccine carrying HIV-1 genes gag, pol and nef of subtype B was in 2007 terminated as the vaccine was shown to be ineffective and even associated with an increased transmission in uncircumcised MSM volunteers with pre-existing adenovirus immunity. At present, combining DNA vaccines and live-vector based vaccines in prime-boost regimens appears to be the most promising vaccine strategy. However, many issues remain to be addressed. One issue is the efficacy of the DNA immunization, which is the topic of this investigation. Another issue is that animal studies so far indicate that this approach might offer a limited protection from infection and also has the potential to lower the viral load post infection and potentially protect from development of disease. A reduction of the viral load of infected individuals could reduce the risk of HIV transmission. The focus on a lowering of the viral load (in contrast to the induction of sterilizing immunity) constitutes a change in paradigm in HIV vaccine development and has profound implications on vaccine efficacy trials.

HIV exists in many subtypes with different prevalence in different parts of the world. It was shown by several groups that infection or immunization with one subtype may induce immunity to a different subtype (Leandersson et al., 2000 and several papers thereafter). The level of conservation of the amino acid sequence between subtypes varies depending on what viral protein is analyzed and it is possible that it is warranted to include sequences from several subtypes for some of the viral proteins (e.g. envelope proteins). For some proteins it might be sufficient to include only one sequence (e.g. the polymerase gene). Today it is prudent to include antigens from the subtypes that are prevalent in the area where the vaccine in planned to be used. In the current trial in Tanzania env of subtypes A and C are included since these are the major subtypes present. Env of subtype B is included since it is closely related to subtype D that also is circulating in Tanzania. Subtype B is the dominating subtype in Europe and the US. Gag of subtypes A and B are included for the same reason. The vaccine is complemented with RT and rev to further broaden the anti-HIV-1 immune responses. The expanded 11 plasmid vaccine also contains nef, tat and protease of subtype B and gag of subtype C. Rev of subtype B is also included to augment the expression of the envelope genes. Env is desirable also in order to raise antibodies. Since it is highly variable it is prudent to try to match it as to the predominating subtypes. Gag and RT are known to give rise to potent CTL responses and are less likely to vary. A larger number of antigens will also increase the likelihood that an individual regardless of HLA type will react to more epitopes. Reactivity to an increased number of (gag) epitopes has been associated with lower viral load in infected vaccine recipients in the STEP study and strong gag-responses has also been shown in HIV-1 patients that are able to control viral replication (Kiepiela et al., 2007).

*Plasmid DNA-vectored genes* have been shown to induce primarily cell-mediated memory immune responses, but requires high doses to immunize humans when delivered im by conventional needle. Immune responses have been successfully boosted by immunogens included in various viral vectors, such as vaccinia, fowlpox or adenoviruses. However the doses of DNA that needs to be given are substantial and it would be a great advantage if a more efficient delivery mode/route can be found. We have recently shown, in HIVIS 01/02, that the proposed HIV DNA vaccine to be used in this study can prime as efficiently when used at a ¼ of the dose when administered intradermally with the Biojector 2000®, compared to intramuscular administration. Despite similar capacity to prime for a subsequent boost of MVA, the i.d. DNA prime alone induced responses in only 3/10 volunteers as compared to 7/9 after the im higher dose administration. Furthermore the id administration required 5 injections per immunization, which is impractical on a larger scale.

*Electroporation* is the use of a transmembrane electric field pulse to induce microscopic pores in the cell membrane. The pores allow macromolecules, such as DNA, to pass into the cell. The electropores are located primarily on the surfaces of cells which are closest to the electrodes. If the electric field pulse has the proper parameters, then the "electroporated" cells recover rapidly (the electropores reseal spontaneously), and the cells stay viable and can produce the vaccine protein. The time for the pathways to form is about one microsecond and the pores reseal within minutes thereafter. The Derma Vax™ Intradermal Delivery System (Roos et al., 2006 and 2009) primarily targets the dermis layer of skin. It utilizes a two-step process where the DNA plasmid is intradermally injected by needle or Biojector. Then an array of 2 rows of 6 needle electrodes of 2 mm in length is pressed against the skin, straddling the injection site and followed by the delivery of less than a second of electric pulses. The two main layers of the skin, the epidermis and dermis, contain numerous resident antigen presenting cells such as Langerhans cells and dermal dendritic cells, making the skin an attractive target for DNA immunization. After intradermal plasmid injection into skin, transfected cells are typically restricted to the epidermis. However, when electroporation is applied after the injection, larger numbers of epithelial, fibroblast and dendritic-like cells are transfected. Because of surface access, and because skin electroporation is relatively painless, this methodology is more suitable for human use than intramuscular electroporation. Several studies have shown pronounced augmentation of cell mediated and humoral responses to various antigens in small animals and non-human primates, with intramuscular or intradermal electroporation (Luckay et al., 2007; Hirao et al., 2007 and 2008; Cristillo et al.,2008; Bråve and Wahren., 2008). Clinical studies with HIV DNA plasmids have been initiated in the US using an intramuscular electroporation device (Vasan, 2009). However, muscular contractions can be very painful and this is avoided by the proposed intradermal mode of electroporation.

At present (January 2010) several clinical studies are ongoing with the use of electroporation: In the US with HIV plasmids, in Sweden with HCV plasmids and with Prostate specific antigen.

In the phase I HIVIS 01 study it was found to be safe to use the seven plasmids. This has also been the experience in the ongoing follow-up phase I/II study, HIVIS 03, in Tanzania. We now aim to improve on the DNA immunogenicity with electroporation to allow us to deliver fewer injections and augment the antibody responses. With this safety record, we are planning to proceed to an EDCTP funded phase II trial, TaMoVac, in Tanzania to study the promising modes of DNA delivery followed by MVA and/or boosted by heterogeneous HIV DNA. The rationale for the use of several subtypes in this trial is as delineated above.

**DNA plasmids**

*Derivation of product to be used in this trial*

The DNA vector derives from pUC8 with a kanamycin resistance gene, hCMV promotor, a poly A linker and an E. coli origin of replication.

*Safety, immunogenicity and challenge models of DNA vaccines with electroporation in animals*

In mice

Inbred strains of mice: C57BL/6 and BALB/C were used to determine immunogenicity since they will develop Th1 and Th2 focussed responses, respectively. Challenge with HIV-1 has been performed in an HIV-1/MuLV pseudovirus system (Boberg et al., 2008). The addition of electroporation has been shown to significantly increase both cellular and humoral HIV-specific immune responses in mice. In terms of the immune responses, which normally require three injections of DNA (without electroporation), this can now be achieved by one or two injections using significantly less DNA (Bråve and Wahren, 2008).We have also shown that in animals primed with DNA plasmids (without the addition of electroporation) it is possible to boost immune responses by delivering DNA using electroporation in a heterologous “DNA prime- DNA boost regimen”. By using electroporation it is also possible to increase immune responses to less immunogenic plasmid constructs and thus broaden the anti-viral immune responses.

In macaques

We have performed two experiments in macaques that have shown the safety, immunogenicity and protective efficacy of a prime-boost vaccine regimen based on plasmid DNA expressing various HIV-1 genes (env, gag, RT, rev, tat, nef) and SIV (SIVmac gag/pol) immunogens followed by MVA expressing the same immunogens (Mäkitalo et al., 2004). In the first experiments we found that intramuscular immunization followed by mucosal immunization with a jet injector induced stronger cellular immune responses and more effective control of SHIV challenge virus (given intravenously) than intramuscular immunization only. All vaccinated monkeys had reduced viral loads compared to controls and one of 8 vaccinated monkeys was completely protected against infection. In the second experiment all monkeys (n=6) were first immunized with DNA vaccine intramuscularly and then mucosally with a jet injector followed by two booster immunizations with MVA vaccine. All monkeys showed good cellular immune responses as measured by the ELISPOT IFN-gamma assay and the lymphocyte proliferation assay. Two weeks after intrarectal SHIV challenge the median plasma viral load was two logs lower in the vaccinated monkeys than in the naive controls. In a recent pilot experiment we immunized 3 macaques intradermally with the 7 plasmid vaccine using the Biojector together with electroporation using the Derma Vax™ electroporation system. The immunizations were well tolerated.

*HIV MVA-CMDR*

The use of live attenuated Modified Vaccinia Ankara virus recombinants as expression vectors for heterologous HIV gene inserts has been shown in many clinical trials to be safe and immunogenic (Moorthy et al., 2003; Jaoko et al., 2008; Dorrell et al., 2007; Peters et al., 2007). In particular, Modified Vaccinia Ankara (MVA)-based recombinants have been shown to induce antibodies and specific cellular immune responses mediated by CTLs to the inserted antigens. The Walter Reed Army Institute of Research/National Institutes of Health (WRAIR/NIH) have produced a live recombinant poxvirus vector, MVA-CMDR, that is genetically engineered to express the following HIV genes: gp150 (Subtype E, CM235), gag, and pol (integrase-deleted and reverse transcriptase nonfunctional, Subtype A, CM240). This live attenuated vector has been shown to be safe and immunogenic in two Phase I protocols, either administered alone (RV158 US, Thailand) or used in a DNA prime/MVA boost immunization strategy in Sweden and Tanzania (Sandström et al., 2008; Bakari et al., 2008 and 2009).

*Risk benefit analyses*

Risks directly associated with injection in the skin is local pain. Pain caused by needlefree injection has previously been described as less than the discomfort caused by a conventional needle injection. Pain associated with electroporation in the skin is experienced as a shortlived pinch. The summarized discomfort associated with injections, electroporation and blood sampling is considered to be reasonable in proportion to the importance of HIV-vaccine development. Both needlefree injections and electroporation increase the uptake of DNA resulting in a relatively high concentration of DNA at the site of injection. It is theoretically possible that the local level of DNA increases the risk for integration at the site of injection. In previously performed studies the risk for integration is estimated to be lower than the frequency of spontaneous natural mutations in somatic cells. In this study it is an advantage that the skin is the target for electroporation and can easily be observed if any local disadvantages occur. We have performed preclinical tests for intergration with the plasmids to be used in this study in conjunction with electroporation and found no reason to suspect that integration will be a clinical problem. Punch-biopsies (3 mm) can be performed at different intervals – a procedure that is rutine in dermatological clinics and associated with a local discomfort at the level of blood sampling. The expected information gained is in reasonable proportion to planned actions. A possibility is that a slow decrease in the local level of DNA might improve the immunological effects of the vaccine. A consequence of the more effective immunization technique can be false positive reactions in routine serological diagnostic HIV-tests. All volonteers in this study will be offered access to additional assays that can distinguish the effects of immunization from those of HIV-infection as well as a certificate stating that this person is vaccinated with a vaccine causing immunological reactivites that might cause false postive reaction in conventional HIV-tests.

**General plan**

**1.1 Objectives**

Primary

Is it safe and tolerable to immunize with seven or eleven DNA plasmids carrying HIV-1 genes with electroporation?

Secondary

1. Is it possible to increase immunogenicity of a DNA vaccine delivered intradermally and to reduce the number of injections needed by using electroporation?
2. Will electroporation improve/alter the quality of the immune responses induced by the HIV-1 DNA plasmids?
3. Is it possible to obtain similar immune reactivity by replacing two intramuscular boosts using MVA with two intradermal injections of HIV-1 DNA plasmids together with electroporation?
4. Can electroporation increase immunogenitcity of all included DNA plasmids to permit mixing of all vaccine plasmids instead of delivering them as two different entities?
5. Will additional DNA plasmids broaden the immune response?

**1.2 Design**

The trial is randomised and placebo controlled.

Study Design

| **Group** | **N** | **Mode of Immunization**  **(w 0, 6 , 12)** | **Mode of delivery** | **Left arm (Ampoules 1**  **env)** | | **Right arm (Ampoules 2**  **gag, Rtmut)** | **Boost immunization**  **(w 36 and 60)** |
| --- | --- | --- | --- | --- | --- | --- | --- |
| B1 | 10 | Id  ZetaJet® | No electroporation | 1 inj 100µl  0,3 mg DNA* | 1 inj100µl  0,3 mg DNA* | | 1 im inj needle  MVA 10^8^ pfu |
|  | 2 | Id  ZetaJet® | No electroporation | 1 inj 100µl saline | 1 inj 100µl saline | | 1 im inj needle  100µl saline |
| B2 | 10 | Id  ZetaJet® | electroporation | 1 inj 100µl  0,3 mg DNA* | 1 inj 100µl  0,3 mg DNA* | | 1 im inj needle  MVA 10^8^ pfu |
|  | 2 | Id  ZetaJet® | electroporation | 1 inj 100µl saline | 1 inj 100µl saline | | 1 im inj needle  100µl saline |
| B3 | 10 | Id  ZetaJet® | electroporation | 1 inj 100µl  0,3 mg DNA* | 1 inj 100µl  0,3 mg DNA* | | 2 id inj Zetajet®,  tot. 0,6 mg DNA + electroporation |
|  | 2 | Id  ZetaJet® | electroporation | 1 inj100µl saline | 1 inj100µl saline | | 2 id inj Zetajet®,  tot 200µl saline +  electroporation |
| B4 | 10 | Id  ZetaJet® | electroporation | 1 inj100µl  0,3 mg DNA** mixed ampoules | 1 inj100µl  0,3 mg DNA** mixed ampoules | | 1 im inj needle  MVA 10^8^ pfu |
|  | 2 | Id  ZetaJet® | electroporation | 1 inj100µl saline | 1 inj100µl saline | | 1 im inj needle  100µl saline |

*) 7 DNA plasmids **) 11 DNA plasmids.

Immunizations will be given as prime injections (HIV DNA) at weeks 0, 6 and 12 and as boost injections (MVA) at week 36 and week 60.

**1.3 Population**

The total study population consists of 48 healthy adults not infected by HIV. Subjects will be men or women 18 to 40 years of age. Volunteers must not be engaging in high-risk behaviour for HIV (i.e., injecting drug or sex with HIV positive partner) and must avoid other sexually transmitted infections. Participants will agree to practice effective contraception from study entry until 4 months after the last immunization. For men this will mean consistent condom use and encouragement for partners to use oral contraception or IUD and for women mandatory oral contraception, of IUD or equivalent contraception in addition to consistent condom use. Subjects will be followed up for 12 weeks after the last immunization and will then have their study termination visit according to the protocol schedule of events. Both genders agree to practice safer sex to avoid sexually transmitted infections. Healthy volunteers will be recruited among the general public, medical students, local hospital staff. Advertisements in posters, lectures as well as on the Internet are the main instruments to reach potential volunteers.

### *1.3.1 Inclusion Criteria*

- Men and women 18 to 40 years of age
- Negative antibody/antigen test for HIV infection
- Willing to undergo HIV testing
- Residents in Stockholm, at low risk of HIV and willing to remain so for the duration of the study
- Low risk of HIV infection defined as:

No history of injecting drug use in the previous ten years. No gonorrhoea, chlamydia or syphilis during the last six months. No high risk partner (e.g. injecting drug use, HIV positive partner) either currently or within the past six months.

- Willing to undergo a genital infection screening if need arises
- Participants will agree to practice effective contraception from study entry until 4 months after the last immunization
- Be willing to practice safer sex for the duration of the study to avoid sexually transmitted infections
- Good health as determined by medical history, physical examination and clinical judgment.
- No grade 1 or higher routine laboratory parameters (see section 4.1.4)
- Hb >10.5g/dL
- White blood cell count <13.000/mm3
- Neutrophils >1500/mm3
- Lymphocytes >1.0
- Platelets >120.000/mm3
- CD4 >400/mm3
- Glucose 2.5-7.0 mmol/L
- Bilirubin <1.25xULN
- AST <1.25xUNL
- ALT <1,25xUNL
- ALP <1.25xUNL
- Creatinine <1.0xUNL
- Complete urinalysis (UA). If microscopic UA confirms evidence of hematuria or proteinuria ≥ 1+, the volunteer is ineligible.
- Availability for the duration of the study
- Able to give fully informed consent at screening visits 1 and 2

### *1.3.2 Exclusion Criteria*

- Have active tuberculosis or other systemic infectious process, such as laboratory detection of tuberculosis bacteria, Hepatitis antigen, acute Hepatitis C, acute or active syphilis
- Have a history of immunodeficiency, chronic illness requiring continuous or frequent medical intervention, autoimmune disease, severe eczema
- Have history of psychiatric*,* medical and/or substance abuse problems during the past 6 months that the investigator believes would adversely affect the volunteer's ability to participate in the trial
- History of grand-mal epilepsy, or currently taking anti-epileptics
- Have received blood products or immunoglobulin in the past 3 months
- Are receiving ongoing therapy with immunosuppressive therapy such as systemic corticosteroids or cancer chemotherapy
- Have used experimental therapeutic agents within 30 days of study entry
- Have ECG deviations that indicate heart disease or would make interpretation of vaccine induced effect difficult
- Have received any live, attenuated vaccine within 60 days of study entry. NOTE: Medically indicated subunit or killed vaccines (e.g., Hepatitis or influenza) are not exclusionary but should be given at least 2 weeks before or after HIV immunization to avoid potential confusion of adverse reactions.
- Have previously received an HIV vaccine
- History of severe local or general reaction to vaccination defined as

**Local**: extensive, indurated redness and swelling involving most of the antero-lateral thigh or the major circumference of the arm, not resolving within 72 hours

**General**: fever >= 39.5^o^C within 48 hours; anaphylaxis; bronchospasm; laryngeal oedema; collapse; convulsions or encephalopathy within 72 hours

- Are study site employees who are involved in the protocol and may have access to the immunogenicity results
- Unlikely to comply with protocol

**1.4 Trial products**

*1.4.1 DNA*

Groups B1 - B3: DNA plasmids carrying seven HIV-1 genes; pKCMVgp160A, pKCMVgp160B, pKCMVgp160C, pKCMVp37A(ba), pKCMVp37B2, pKCMVRTmut, pKCMVrev,

Group B4: DNA plasmids carrying 11 HIV-1 genes; pKCMVgp160A, pKCMVgp160B, pKCMVgp160C, pKCMVcoPR, pKCMVRTmut, pKCMVp37A(ba), pKCMVp37B2, pKCMVp37C(bc), pKCMVrev, pKCMVnef, pKCMVtat.

1.4.1.1 Supply, storage and composition of DNA

Vecura will be responsible for bulk manufacture of the clinical material, release testing and technical release of vialed product, and labelling. All procedures will be according to Good Manufacturing Practice.

1.4.1.2 Preparations prior to use of DNA

Prior to use the vials will be thawed at room temperature. When completely thawed, the vials should be gently swirled. Care must be taken not to shake or invert the vials.

1.4.1.3 Labels

DNA will be packaged by Vecura. The trial products will be in vials pre-labelled according to standardised operating procedures. Each vial will be packaged in a box labelled with the name of the clinical site and principal investigator, the storage details and the name of the supplier for the product.

For intradermal use

**For clinical use only. Clinical Trial: HIVIS Env DNA / Ampoule 1 i.d.**

PI: Bo Hejdeman, South Hospital, Stockholm

Sponsor: Swedish Institute for Infectious Disease Control.

Batch#: xxxx, 0.15 ml pKCMV(rev, envA,B,C), 3mg/ml DNA for injection

Expire date: xxxx, Storage: -20 degr C

Man.by: Vecura, Karolinska University Hospital, Sweden

**For clinical use only. Clinical Trial: HIVIS Gag DNA / Ampoule 2 i.d.**

PI: Bo Hejdeman, South Hospital, Stockholm

Sponsor: Swedish Institute for Infectious Disease Control.

Batch#: xxxx, 0.15 ml pKCMV(gagA,B, RTmut) 3 mg/ml

Expire date: xxxx, Storage: -20 degr C

Man.by: Vecura, Karolinska University Hospital, Sweden

**For clinical use only. Clinical Trial: HIVIS-11 DNA / Ampoule 3 i.d.**

PI: Bo Hejdeman, South Hospital, Stockholm

Sponsor: Swedish Institute for Infectious Disease Control.

Batch#: xxxx, 0.35 ml pKCMV(rev, envA,B,C, gagA,B,C,RTmut, nef, tat, PR), 3mg/ml DNA

Expire date: xxxx, Storage: -20 degr C

Man.by: Vecura, Karolinska University Hospital, Sweden

Boxes will be supplied to the pharmacist for storing the used vials, labelled with the supplier, the name of the trial and protocol version; the participant study number; and the name of the clinical site and principal investigator.

1.4.1.4 DNA delivery

**ZetaJet®.** Each id ZetaJet® inoculation consists of 100 ul (300 ug of DNA). DNA is thawed according to provided SOP, filled in ZetaJet® and 100 ul applied intradermally to skin of one arm. Directly after the administration of DNA, the **DermaVax™** electroporation needles are fitted to straddle the id swelling and a short (1/3 second) train of electric pulses given.

*1.4.2 MVA and delivery*

1.4.2.1 Supply, storage and composition of MVA

Vials of prelabelled HIV MVA-CMDR with a concentration of 10^8^ pfu/mL are received by SMI from WRAIR, for delivery to Apoteket, Södersjukhuset. The storage temperature is -80 C. The MVA-CMDR vaccine is handled in accordance to previous clinical trials at Venhälsan, SÖS. The vials containing, 1,2 mL (10^8^ IU/ml), are stored at SMI in -80ºC and transported to Apoteket, Södersjukhuset, prior to each vaccination.

1.4.2.2 Preparations prior to use of MVA

The vials are thawed to room temperature, and 1.0 mL (10^8^ pfu) given intramuscularly according to a separate SOP. The vaccine is filled in a syringe, packed at Apoteket, Södersjukhuset and transported to the clinic.

1.4.2.3 Labels

Each vial is for single-dose use and will be given a label containing the following information; PI, vaccine name, dosage, batch number, contents, storage directions, caution warning, manufacturer, and date of manufacturing. Vaccine will be administered by intramuscular injection (IM, 1.0 mL) in the deltoid muscle.

1.4.3 Dispensing records and disposal of unused product

The designated pharmacist will, upon receipt of supplies prior to commencement of the trial, conduct an inventory and complete a receipt, one copy of which will be retained at the site, and the original returned to the supplier. During the trial the pharmacist will be responsible for reviewing the dispensing log.

On the day prior to immunisation the study nurse will request the study number, date of birth, and immunisation number, and transfer vaccine for use the following day to the clinic without breaking the cold chain. The immunisation number and date will be entered against the study number in the dispensing log. The vial label will be crosschecked against the details on the prescription and dispensing log by two individuals, prior to product being administered.

The individual who administers the injection will be responsible for ensuring that the return of the used vials is recorded in the dispensing log at the end of the clinical session, and that they are placed in the appropriate participant box.

At the end of the trial all used and unused vials will be checked against the inventory by the monitor before disposal on site according to local pharmacy guidelines and applicable regulations. Documentation of disposal will be provided to the monitor and supplier.

During the trial, product accountability will be monitored by the prescriptions, the dispensing log, the returns, the trial register and data collected on the case report forms.

**1.5 Endpoints**

*1.5.1 Primary:*

1. The safety of immunization will be assessed by clinical signs and standard biochemical and haematological laboratory tests where any worsening of the severity grade will be considered for causality with the vaccine.
2. The immunization will be evaluated by assessing local (pain, cutaneous reactions including indurations), general (fever, chills, headache, nausea, vomiting, malaise, myalgia) and other unsolicited adverse events within 28 days. Any grade III or IV event will be taken as an indication that the group of patients tolerate this schedule less well.

The primary safety parameters will be graded:

- Local adverse event Grade 3 or above (pain, cutaneous reactions including indurations).
- Systemic adverse event Grade 3 or above (temperature, chills, headache, nausea, vomiting, malaise, or myalgia).
- Other clinical or laboratory adverse events Grade 3 or above confirmed at examination or on repeat testing.

Any event attributable to vaccine leading to discontinuation of the immunisation regimen must be documented. Data on local and systemic events listed above will be solicited with specific questions or using a diary card for 7 days following each immunisation. Data on other clinical events and laboratory events will be collected with an open question at each visit and through routinely scheduled investigations.

*1.5.2 Secondary:*

A qualitative evaluation of cellular responses against peptides representing the different immunogens will be performed primarily by the ELISPOT technique. Additional testing will be performed by intracellular cytokine staining quantifying IFN-γ, IL-2, TNF-α and MIP-1β production by CD4 and CD8 T cells, lymphoproliferation against inactivated HIV using fresh cells and multi-colour intracellular cytokine staining using cryopreserved cells for assessment of multiple T cell effector functions including immunophenotyping of responding cells. Testing for binding antibodies and, if binding antibodies are present, also neutralizing antibodies will be performed.

Group B1 will be compared with the id only group in the HIVIS study (Group A in HIVIS trial) for evaluation of the lower dose of HIV DNA (0.6 vs 1.0 mg).

Group B2 will be compared with Group B1 for evaluation of the lower dose of HIV DNA with and without electroporation.

Group B3 will be compared to the group B2 for the evaluation of HIV DNA as a boost compared to boosting with HIV MVA.

Group B4 will be compared to groups B1-B3 to show if priming with additional HIV-related plasmids will increase immunogenicity to any of the included antigens.

###

### 2 Schedule of Visits

**2.1 Recruitment**

Healthy volunteers will be recruited among the general public, medical students and local hospital staff. Advertisements in posters, lectures as well as on the Internet are the main instruments to reach potential volunteers.They will be provided with further information about the study, and asked to complete a short interview (by telephone or in person) to assess their suitability. At this point they will be given a personal identification four-digit study Medical Record Number (MRNO). They will be given or sent an information sheet about the trial.

If they are still interested and willing to participate, they will be invited to attend screening.

**2.2 Screening**

The **first screening visit** (study visit 1) will take place between one to four weeks before visit two. At this visit the study subject will meet the study doctor(s) and study nurse(s) and the trial will be discussed in detail and an Informed consent for screening signed. The study doctor will take a complete medical history and perform a physical examination including mouth and throat inspection, palpation of lymph nodes, cardiac and pulmonary auscultation, abdominal palpation, blood pressure and body temperature. Medical status and medical history, including any ongoing medication and any previous allergic reactions, will be documented. These investigations can be postponed to the following visit. To ensure informed consent, subjects will go through the following process with a member of the study team and a more detailed check of eligibility conducted using a case record form and any questions about the study will be answered.

1. Pre-HIV test counselling
2. Safer sex counselling
3. That they should continue to use condoms with sexual partners whose HIV status is not known
4. The meaning of ‘randomisation’
5. The information will include potential adverse effects of the vaccine
6. Information about the fact that it is unknown whether or not the study vaccines will protect against HIV infection
7. Information that following immunisation they may develop antibodies that might produce a positive reaction in a routine HIV test, but that provisions have been made to distinguish between a post vaccination response and HIV infection during and after the trial
8. That participants probably will be excluded from donating blood or organs
9. That they may be subject to social risk if they develop HIV antibodies, or by revealing their participation in the study
10. The level of care that will be made available to them if they should be found to be HIV infected at any time during their participation in the study, including the screening period.

After signing written study consent the study subject is allowed to donate blood for safety laboratory tests including HIV antibody/antigen test. After a volunteer is found to be eligible, the screening number will become the study number. This means that study numbers may be discontinuous. The visit should be recorded in the source documents including conformity to inclusion and exclusion criteria, study number and any other information relevant to inclusion in the study.

**2.3 Trial Entry**

The **second screening visit** (study visit 2) takes place one to four weeks before the first planned injection. A member of the study team will confirm that the volunteer is at low risk of HIV-1 infection, that all inclusion criteria are fulfilled (see section 1.3.1), that no exclusion criteria exist (see section 1.3.2) and the volunteer understands and agrees to the concept of practicing effective contraception. If not already done at the prior visit, the study doctor will take a complete medical history and perform a physical examination including mouth and throat inspection, palpation of lymph nodes, cardiac and pulmonary auscultation, abdominal palpation, blood pressure and body temperature. Medical status and medical history, including any ongoing medication and any previous allergic reactions, will be documented. The volunteer will again receive an extended information, both oral and in written, describing the study.

The volunteer will also be informed that s/he is free to end all obligations in the study whenever s/he wishes and that a premature ending will not have any negative influence in future contacts with the clinic. At this visit the study nurse confirms that the volunteer fulfils all the inclusion criteria, including laboratory results, makes an abbreviate risk assessment and a brief summary of the study design. Reassurance has to be made that the study subject fully understands the concept of the study. A mobile/telephone number and name of contact is obtained for the follow up. The results of the screening investigations will be reviewed and volunteers who are still eligible and willing will be asked to complete part 2 of the Informed consent. Blood is donated according to flow chart and an ECG evaluation will be performed.

The visit should be recorded in the source documents including conformity to inclusion and exclusion criteria, study number and any other information relevant to inclusion in the study.

2.4 Randomisation and study procedures

At **week 0 (study visit 3)** the study subject again meets both study nurse(s) and study doctor(s). Once again confirmation has to be made that the volunteer fulfils all inclusion criteria, including laboratory results, and fully understands the concept of the study. If no contraindications are revealed the study subject is randomised into one of the three study groups (B1 – B3) in the first part of the study and to group B4 in the second part of the study and again blood is donated for safety laboratory tests. The randomisation procedure is documented. The individuals can participate in the trial without consent to genetic analyses.

A randomization list comprising the 48 vaccination assignments will be prepared by an independent statistician in two sets, one of 36 (B1-3) and one of 12 (B4). Labels with the corresponding assignments will be placed in numbered two serials of envelopes (one for groups B1-3 and another for group B4) by the pharmacist and sealed. The randomization schedule will be kept under lock by the pharmacist and the envelopes delivered to the clinic. In case of drop-outs prior to the first immunization the labels will be returned to the pharmacist who will undertake a second randomization at the end of the recruitment. Drop-outs after the end of the recruitment will not be replaced. After randomization blood is donated according to flow chart and the first injection is given. The study visit is documented. Volunteers will be observed for 30 minutes following injection for acute adverse experiences and will also be contacted the day following injection by the study nurse for a brief adverse reaction interview. The number given for the follow-up telephone contact and the contact (in case the volunteer is not reached) is checked as current. In addition, volunteers will complete diaries over events for 7 days following each vaccination and receive telephone numbers to call, all hours of the day for 7 days after the vaccination, in order to get in contact with the clinic if needed. A folder with diary cards, thermometer and a ruler will be issued to each patient. The visit should be recorded in the source documents including immunization, study number and any other information relevant to the study.

**Week 2 (study visit 4)** is conducted by the study nurse(s). The study form is filled in and blood donated according to flow chart. Abbreviated risk assessment and risk behaviour counselling is done. The visit should be recorded in the source documents including study number and any other information relevant to the study.

**Week 6 (study visit 5),** the visit for the second injection of the study vaccine, is conducted by both the study nurse and the study doctor. Before injection the diaries on events after the last injection will be discussed with the study subjects and an abbreviated risk assessment and risk behaviour counselling will be performed. Enough time has to be planned for the visit so that all questions from the volunteer about the study can be answered or reflected upon. After blood donation the second injection is given and the visit is documented. Again volunteers will be observed for 30 minutes following injection and new diaries on events will be completed for 7 days. The visit should be recorded in the source documents including immunization, study number and any other information relevant to the study.

**Week 8 (study visit 6)** is conducted by the study nurse(s). The study form is filled in and blood donated according to flow chart. Abbreviated risk assessment and risk behaviour counselling are done. The visit should be recorded in the source documents including study number and any other information relevant to the study.

**Week 12 (study visit 7),** the visit for the third injection of the study vaccine, is conducted by both the study nurse and the study doctor. Before injection the diaries over events after the last injection will be discussed with the study subjects and an abbreviate risk assessment and risk behaviour counselling will be performed. Enough time has to be planned for the visit so that all questions from the volunteers about the study can be answered or reflected upon. After blood donation the third injection is given and the visit is documented. Again volunteers will be observed for 30 minutes following injection and new diaries over events will be completed for 7 days. The visit should be recorded in the source documents including immunization, study number and any other information relevant to the study.

**Study visits 8, 9 and 10** planned at weeks 14, 16 and 32 respectively. They are all conducted by the study nurse(s). At all visits the study forms are fulfilled and blood donated according to flow chart. Abbreviated risk assessment and risk behaviour counselling are planned for all visits except at visit 4 (two weeks after the last injection) when only blood tests and discussion over “event diaries”, fulfilled after the second injection, are planned. However at all time points enough time has to be planned in order to answer questions from the volunteers. Optionally at visit 8 or 9 a 3 mm punch biopsy will be taken from the site of the electroporated immunization site. At visit 10, eligibility for the next phase of immunization will again be checked.

**Week 36 (study visit 11)**, the visit for the forth injection of the study vaccine, is conducted by both the study nurse and the study doctor. Before injection the diaries over events after the last injection will be discussed with the study subjects and an abbreviate risk assessment and risk behaviour counselling will be performed. Enough time has to be planned for the visit so that all questions from the volunteers about the study can be answered or reflected upon. After blood donation the third injection is given and the visit is documented.

Again volunteers will be observed for 30 minutes following injection and new diaries over events will be completed for 7 days. The visit should be recorded in the source documents including immunization, study number and any other information relevant to the study.

**Week 38 (study visit 12)** is conducted by the study nurse(s). The study form is filled in and blood donated according to flow chart. Abbreviated risk assessment and risk behaviour counselling are done. The visit should be recorded in the source documents including study number and any other information relevant to the study.

**Week 40 (study visit 13)** is conducted by the study nurse(s). Blood is donated according to flow chart.

**Week 60 (study visit 14)** the visit for the fifth and last injection of the study vaccine, is conducted by both the study nurse and the study doctor. Before injection the diaries over events after the last injection will be discussed with the study subjects and an abbreviate risk assessment and risk behaviour counselling will be performed. Enough time has to be planned for the visit so that all questions from the volunteers about the study can be answered or reflected upon. After blood donation the fifth injection is given and the visit is documented. Again volunteers will be observed for 30 minutes following injection and new diaries over events will be completed for 7 days. The visit should be recorded in the source documents including immunization, study number and any other information relevant to the study.

**Week 62 (study visit 15)** is conducted by the study nurse(s). The study form is filled in and blood donated according to flow chart. Abbreviated risk assessment and risk behaviour counselling are done.

**Week 64 (study visit 16)** is conducted by the study nurses(s)**.** Blood is donated for immunology.

**Week 72 (study visit 17)** is the final planned visit in the study. The visit is conducted by both the study nurse and the study doctor. Again the study doctor takes a complete medical history and performs a physical examination according to study visit 1. An extended risk assessment and risk behaviour counselling is performed and blood is donated according to flow chart. The visit should be recorded in the source documents including study number and any other information relevant to the study. All study subjects will be issued a card signed by the principal investigators (full names, tel. numbers and addresses) stating that the holder has participated as a volunteer in a HIV-1 immunization study and that the outcome of standard HIV diagnostic tests do not signify HIV-1 infection.

*2.4.1 Immunisations*

| **Group** | **N** | **Mode of Immunization**  **(w 0, 6 , 12)** | **Mode of delivery** | **Left arm (Ampoules 1**  **env)** | | **Right arm (Ampoules 2**  **gag, Rtmut)** | **Boost immunization**  **(w 36 and 60)** |
| --- | --- | --- | --- | --- | --- | --- | --- |
| B1 | 10 | Id  ZetaJet® | No electroporation | 1 inj 100µl  0,3 mg DNA* | 1 inj100µl  0,3 mg DNA* | | 1 im inj needle  MVA 10^8^ pfu |
|  | 2 | Id  ZetaJet® | No electroporation | 1 inj 100µl saline | 1 inj 100µl saline | | 1 im inj needle  100µl saline |
| B2 | 10 | Id  ZetaJet® | electroporation | 1 inj 100µl  0,3 mg DNA* | 1 inj 100µl  0,3 mg DNA* | | 1 im inj needle  MVA 10^8^ pfu |
|  | 2 | Id  ZetaJet® | electroporation | 1 inj 100µl saline | 1 inj 100µl saline | | 1 im inj needle  100µl saline |
| B3 | 10 | Id  ZetaJet® | electroporation | 1 inj 100µl  0,3 mg DNA* | 1 inj 100µl  0,3 mg DNA* | | 2 id inj Zetajet®,  tot. 0,6 mg DNA + electroporation |
|  | 2 | Id  ZetaJet® | electroporation | 1 inj100µl saline | 1 inj100µl saline | | 2 id inj Zetajet®,  tot 200µl saline +  electroporation |
| B4 | 10 | Id  ZetaJet® | electroporation | 1 inj100µl  0,3 mg DNA** mixed ampoules | 1 inj100µl  0,3 mg DNA** mixed ampoules | | 1 im inj needle  MVA 10^8^ pfu |
|  | 2 | Id  ZetaJet® | electroporation | 1 inj100µl saline | 1 inj100µl saline | | 1 im inj needle  100µl saline |

*) 7 DNA plasmids **) 11 DNA plasmids.

Immunizations will be given as prime injections (HIV DNA) at weeks 0, 6 and 12 and as boost injections (MVA) at week 36 and week 60.

*2.4.2 Injections*

All **DNA** immunizations will be performed with a needle-free device, ZetaJet®, according to the manufacturer’s (Bioject Inc, Portland, Oregon) instruction.

Intradermal injections will be given over the deltoid muscle. Ampoule 1 will be given in the left arm and ampoule 2 in the right arm, except in the Group B4 where a mixture of vaccines from two identical ampoules will be given in both arms. Immediately after the injection subjects randomized to Groups B2, B3 and B4 will receive intradermal electroporation over the injection sites with the CytoPulse device, DermaVax, according to the manufacturer’s instruction.

All **MVA** injections will be performed with needle and syringe in the left deltoid muscle.

The site will be inspected after the immunisation, and any local reactions recorded on the case record form.

The immunisations will take place in an outpatient setting. Participants will be closely observed for 30 minutes after each immunisation, at which point vital signs (pulse, blood pressure and respiratory rate) will be recorded on the case record form, as well as any local or systemic reaction.

The needles, syringes and other materials will be disposed of according to local hospital regulations.

As with any parenteral vaccine, epinephrine and corticosteroids must be available for immediate use if an immediate hypersensitivity reaction, such as anaphylaxis, occur. Intravenous injections of vaccines will NOT be performed.

Study vaccine must not be administered to individuals with hypersensitivity to any component of the vaccine.

*2.4.3 Safety follow-up in the days following immunisation*

A diary card will be given, with instructions and a full verbal explanation, for participants to record local and systemic adverse events following immunisation, as well as medication taken. Volunteers will also be contacted the day following injection for a brief adverse reaction interview and subjects reporting any grade 2 or higher event will be prompted to a clinic visit. Should the volunteer be unreachable the designated contact will be contacted. In addition, volunteers will complete diaries over events for 7 days following each vaccination. Contact will be maintained by the clinical team, using a method chosen by the participant, as indicated by the evolution of adverse events, up to resolution of solicited local and systemic events. Additional visits may be recommended at the discretion of the clinical and principal investigators, if clinically indicated or in order to clarify observations.

All concomitant medication must be recorded in study form CRF with the reason for administration, the dosage regimen, the onset and end of treatment.

The following medications are prohibited while the subject is in the study:

- Immunomodulatory agents (i.e. immunoglobulin)

- Immunosuppressive agents (i.e., systemic steroids, chemotherapy)

- Live, attenuated vaccines. Other vaccines (subunit or killed) should be given at least two weeks before or two weeks after HIV immunization.

**2.5 Final visit**

Assessments will be undertaken according to schedules in 2.6.

*2.5.1 Reimbursement*

Regular payments will be made to all participants to cover their travel expenses and any inconvenience caused. At the end of the study all participants will have received remuneration of 5.000 SEK if they participated in the whole study. If they did not participate in the whole study they will receive the respective proportion of remuneration. Those who have to attend for additional visits will be reimbursed accordingly. During the study participants will receive latex condoms free of charge. If any medication is required as a result of the study, this will also be provided free of charge.

*2.5.2 In the event of discontinuation*

A subject may withdraw his/her consent to participate in the study at any time without prejudice or for other reasons, see section 3.7. Whenever possible, the tests and evaluations listed for the termination visit should be carried out if the subject refuses follow-up according to the protocol visit schedule. The DSMC should be notified of all study withdrawals within 48 hours. Subjects who withdraw from the study will be replaced only as long as the study is still open for enrolment. No replacement will be allowed when the enrolment is closed. All subjects who receive at least one immunization will be included in the safety analysis. If a subject does not complete the immunization schedule following a serious adverse event or toxicity, he or she will continue to be followed according to the protocol visit schedule, and, as a minimum, until the adverse event/toxicity is resolved and/or the cause is identified. A genuine effort will be made to determine the reason(s) why a subject fails to return for the necessary visits. If the subject is unreachable by telephone, at least one registered letter will be sent to the subject requesting contact with the clinic. This information will be recorded on the appropriate source document.

**2.6 Procedural Flow Chart**

| Study Visit | 1 | 2 | 3 | 4 | 5 | 6 | 7 | 8 | 9 | 10 | 11 | 12 | 13 | 14 | 15 | 16 | 17 |
| --- | --- | --- | --- | --- | --- | --- | --- | --- | --- | --- | --- | --- | --- | --- | --- | --- | --- |
| Week | a | b | 0 | 2 | 6 | 8 | 12 | 14 | 16 | 32 | 36 | 38 | 40 | 60 | 62 | 64 | 72 |
| Recruitment, medical record number, Screen form 1  Study Consent  Risk Assessment  Study Consent 2  Abbreviated risk assessment | X  X  X | X  X |  |  |  |  |  |  |  |  |  |  |  |  |  |  |  |
| Immunizations |  |  | X |  | X |  | X |  |  |  | X |  |  | X |  |  |  |
| Complete History / Physical examination *Including weight(kg), height (cm),*  *arm circumf.(cm)*  Interim H&P (AE elicitation)  Risk behaviour counselling  Randomisation  ECG  *Genital swabs or urine for Neisseria gonorrhoeae, and chlamydia trachomatis will be collected if indicated according to clinical signs and symptoms* | X  X | X | X  X | X | XX | X | XX | X | XX | X  X |  |  | X  X |  | X  X |  | X  X |
| Punch biopsy* |  |  |  |  |  |  |  | X | X |  |  |  |  |  |  |  |  |
| HIV antibody/antigen test  Syphilis, HBV & HCV  CD4 counts  HIV RNA / DNA PCR*  WBC with differential  Chem. Pathology  Troponin I  Urine analysis; urine dipstick for protein/blood  Urine test of female volunteers for pregnancy (UPT)  Urine, saliva, semen, vaginal and rectal wash* | X  X  X  X  X  X  X  X | X  X  X  X | X  X | X  X  X | X  X  X  X | X  X  X | X  X  X  X | X  X  X  X | X  X  X  X  X  X | X  X  X | X  X | X | X  X  X | X  X | X  X  X  X  X |  | X  X  X  X  X  X |
| Binding and neutralizing antibodies, incl. Anti-vaccinia Ab (red top)  IFN-gamma ELISPOT, fresh cells (Na-heparin, green top)  8-colour ICS, fresh cells (Na-heparin, green top)  Lymphoproliferation assay, fresh cells (Na-heparin, green top)  Multi-colour ICS, cryo-preserved cells (Na-heparin, green top)  Cryo-preserved cells* (Na-heparin, green top)  HLA analysis* (EDTA, purple top)  *Volume for immunogenicity*  **)optionally* |  | X  X  X  X  X  X  *100* | X  (X)  (X)  (X)  X  X  X  *100* |  |  | X  X  (X)  X  X  X  *100* | X | X  X  (X)  X  X  X  *100* | X | X | X  X  X  X  X  X  *100* | X  X  (X)  X  X  X  *100* | X  X  X  (X)  X  X  *100* | X  X  X  X  X  X  *100* | X  X  (X)  X  X  X  *100* | X  X  X  (X)  X  X  *100* | X  (X)  (X)  (X)  X  X  *80* |
| TOTAL BLOOD VOLUME (ml) | 60 | 110 | 110 | 10 | 10 | 110 | 20 | 110 | 35 | 20 | 100 | 100 | 110 | 100 | 110 | 100 | 110 |

## 3 Procedures

**3.1 Blood and urine collection**

Blood will be collected using a sterile needle, usually from the ante-cubital fossa, according to the schedule and transported to the appropriate laboratories. Urine will be collected into a sterile container as indicated on the schedule and either transported to the appropriate laboratory or tested by a member of the clinical team according to trial specific standardised operating procedures. The optional collection of saliva (spitting or collection in a swab), vaginal secretions (by an absorbent device) or rectal washings (instillation of 200 ml NaCl) will be performed according to a standardized protocol and frozen.

The total estimated volume of donated blood is estimated to 1325 ml during the study; approximately 76 weeks.

- All biological samples to be analysed by the clinical laboratory will be collected and processed according to institutional guidelines and regulations regarding biobanks.

- All samples sent to the laboratories at the Swedish Institute for Infectious Disease Control will be labelled with the following information

Medical record number: Subject initials:

Study day: Visit number:

Date

Type of sample (serum, plasma, blood etc)

All samples for routine haematology and biochemistry will be labelled according to hospital rules with the full study subject identity.

Samples that will be analysed after freezing should be prepared, labelled, and stored at the appropriate temperature. Specimens that are shipped will be correctly prepared, labelled, and kept at the correct temperature.

**3.2 Clinical history and examination**

A past medical history will be collected at the screening visit, including details of any previous reaction to vaccination, history of epileptic fit, exposure to vaccinia, and contraceptive and smoking practices. The general examination will include weight (kg), height (cm), blood pressure, and inspection of the skin to exclude severe eczema and checked for the presence of an electroporation scar, a vaccinia scar, respiratory, cardio-vascular and abdominal systems examination. An assessment of cervical and axillary lymph nodes will also be undertaken. The examination results and history of exposure to vaccinia will be recorded on the case record form.

**3.3 Genital infection screen**

The following will be collected in all participants

- serology for syphilis
- markers of hepatitis B and hepatitis C infection

The following will be collected if indicated on account of risk and symptoms, according to trial specific clinical standardised operating procedures:

- urethral, vaginal, cervical and rectal specimens as appropriate for Neisseria gonorrhoeae
- collection of urine for Chlamydia trachomatis

**3.4 Pre-HIV test screen and counselling**

Study personnel will assess volunteers for past and current risk of HIV infection using the screening proforma and counsel them prior to collecting blood for an HIV test. The counselling process will ensure that volunteers have sufficient knowledge about HIV infection to understand what the test is for, the implications of a positive, negative and equivocal result and the standard of care available for HIV infection locally. They will also be informed how and when they will receive the result, according to the local policy in each clinical centre.

**3.5 Safer sex counselling and condom provision**

Participants will be counselled by study personnel about the importance of condoms at screening and reminded on the day of each immunisation. Hypo-allergenic condoms will be provided free of charge to participants throughout the trial.

**3.6 HIV related issues**

Please see section 6 for details of the procedures that may be required in the event of:

- A request for an HIV test
- HIV infection
- Social discrimination as a result of post-vaccine response
- The possibility of a false positive test result in a standard HIV test

**3.7 Discontinuation procedures (including withdrawal and stopping the study)**

Participants may withdraw at any time if they wish to do so, for any reason. The date of withdrawal and reason for doing so should be recorded in the appropriate case record form.

The clinical investigator may decide that it is not in the best interests of the participant to proceed to the next immunisation following an adverse event (see below). The discontinuation and reason should be recorded in the appropriate immunisation and reportable adverse event case record forms, which should be forwarded to DSMC and sponsor within the next 2 working days. Trial visits should carry on at least until resolution or stabilisation of the event, but ideally up to the last visit in the schedule, provided the participant is willing. The frequency of visits and laboratory investigations may be reduced or increased on consultation with the Principal Investigator or Trial Management Group (see section 7).

*3.7.1 Criteria for withdrawal of subjects from injection schedule*

Under certain circumstances, a subject will be terminated from participating in further injections. These events include:

1. HIV infection.
2. Grade 2 events classified as probably associated with immunization that in the view of the clinician or the volunteer are unacceptable.
3. Grade 3 or 4 adverse events classified as probably associated with immunizations.
4. Type 1 hypersensitivity associated with immunization.
5. Serious intercurrent illness that is not expected to resolve prior to next scheduled immunization.
6. Any condition that could put the volunteer at risk or hamper the evaluation of subsequent immunizations.
7. Need for systemic glucocorticoids or other immunomodulators than NSAIDs for any reason.
8. Repeated failure to comply with protocol requirement.
9. The Sponsors, vaccine provider or principal investigator decide to stop or cancel the study.
10. The ethics committee or Medical Products Agency request that the study be stopped.

Patients who are discontinued from additional study injections will continue to be followed according to the schedule to further evaluate safety and monitor adverse events.

*3.7.2 Criteria for stopping the study*

The Principal investigator will closely monitor and analyse study data as they become available and will make determinations regarding the presence and severity of the adverse events. The administration of study injections and new enrolments will be halted if one subject develops a grade 3 event assessed as, possibly, or probably related to the vaccination or experiences a grade 4 or 5 (death) SAE assessed as possibly, related to the vaccination. Safety data and changes in study status should be submitted to the ethics committee in case the study is stopped. In the case that the trial is stopped or halted, this will be reported to the MPA by using the form obtained from the EudraCT system.

**3.8 Unblinding procedure**

The PI or his deputy may ask the monitor to unblind an individual who develops a grade 3, 4 or 5 (death) SAE assessed as possibly related to the vaccination if it is in the volunteers best interest. The monitor may be asked to compile interim data for example for the regulatory authorities in Tanzania. Such data should remain blinded to the study staff and participants, but should be supplied to the DSMC. The DSMC will not be demanded to act on such data unless there are safety issues. When all volunteers have finished the DNA immunizations the monitor and statistician will compile group safety and immunological data while study staff and volunteers remain blinded.

**4 Assessments**

**4.1 Safety assessments**

The subject will be contacted the day after each immunization by the investigator, study nurse, or research assistant to obtain local and systemic reaction data and to determine the subject's clinical status.

Subjects will be instructed to call the specified study personnel immediately if any unusual or severe sign or symptom appears after immunization. These subjects should be seen in the clinic at the time of maximal symptoms, if possible, and will be followed up clinically until resolution of symptoms.

An adverse event is any undesired, noxious or pathological change in a patient or subject as indicated by physical signs, symptoms, and/or laboratory changes that occurs following administration of one of the vaccines, whether or not considered vaccine related. This definition includes intercurrent illnesses or injuries, and exacerbation of pre-existing conditions. The Steering Committee will arrange for a compilation of safety data when 10 subjects have received the electroporation. These data will be reviewed by the monitor for accuracy and submitted to the DSMC and Sponsor.

*4.1.1 Local adverse events*

Following the intradermal injections the site will be graded as dry, moist or wet. The presence or absence of a weal will be recorded. The pain will be recorded as none (0), mild (1), moderate (2), severe (3) or extreme (4). Following the electroporation the pain will be recorded as none (0), mild (1), moderate (2), severe (3) or extreme (4). Following the MVA pain in the muscle injected will be graded by the participant as none (0), mild (1), moderate (2), severe (3) or extreme (4).

This will be recorded in the appropriate case record form or once the participant has left the clinic, in the diary card.

Redness will be recorded as the maximum diameter in cm and graded on this criteria and the presence of symptoms according to the appended scale. Participants will be asked to record any discomfort and any medication taken for relief of symptoms.

Clinical staff will complete the case record form following each immunisation. Completed caserecord forms and the diary cards will be sent to the study nurse at Venhälsan to be recorded in the database. In the event that there are two observations on the same day, those confirmed at a visit will take precedence over diary card observations in the analysis.

Study personnel should verify all grade 3 or 4 events and record the findings in the source documents.

*4.1.2 Systemic adverse events*

Temperature will be measured by the oral route prior to immunisation and 30 minutes after the immunization by study personnel, and graded according to the appended scale. Participants will be given thermometers to record their temperature in the diary card on the evening of immunisation, and daily thereafter for 7 days, and if still raised, they will be advised to continue to monitor their temperature daily until it returns to normal. The temperature observed by the clinical team will be recorded on the case record forms for visits.

Chills, headache, nausea, vomiting, malaise and myalgia will be graded by the participants, recorded in the diary card on the evening following immunisation and daily for 7 days, or until resolution of symptoms whichever is longer. On days where a visit coincides with the diary card, the clinical staff will collect the information directly and record it in the case record form, and this will take precedence over diary card entries in the analysis.

Study personnel should verify all grade 3 or 4 events and document the findings in the source documents.

*4.1.3 Other adverse events*

These will be recorded as reported following an open question to participants, with the dates of commencement and resolution, and any medication required. They will be graded according to the general principles outlined. Social harm will also be recorded as an adverse event, graded according to the general guidelines.

Where a diagnosis is possible, it is preferable to report this rather than a series of symptoms relating to the diagnosis. When reporting a syndrome, indicate the associated signs and symptoms parenthetically following the syndrome rather than as separate events.

Study personnel should verify all grade 3 or 4 events and document the findings in the source documents.

*4.1.4 Routine laboratory, HIV serology and urinary parameters*

The following safety assessments will be undertaken in laboratories local to the clinical centre according to standard procedures subject to quality control.

Haematology: haemoglobin, white cell and platelet count, neutrophils and lymphocytes

Chemical pathology: liver function tests (AST/ALT, alkaline phosphatase, bilirubin), creatinine,

glucose

Immunology; CD4 number and percentage

HIV diagnostic testing: samples will be tested at SMI using ELISA and Western blot according to standard procedures subject to quality control

HIV serology for seropositivity will be performed as standardized for the clinic before and at the end of the study. In case of positive reactions, samples will be analyzed starting from the latest sampling and suspect positive reactions verified by PCR (viral load) at SMI according to standard procedures subject to quality control.

A mid-stream urine specimen will be collected for analysis.

Adverse events that are grade 3 and 4 or necessitate a physician's visit or a prescribed medication will be evaluated. The subject should be followed carefully until the condition is resolved and/or the cause is identified. Any medication or other therapeutic measure taken to relieve symptoms of the medical problem must be recorded on the appropriate case report form page(s) and in the source documents in addition to the outcome of the adverse event.

**4.2 Immunogenicity assessments**

*4.2.1 Antibody responses*

Detection of binding antibodies to HIV proteins covering the vaccines will be assessed using ELISA.

Optional assay

Analysis of neutralizing antibodies to HIV subtypes A, B, C and E in case of existing binding antibodies.

*4.2.2 Cellular responses*

The following HIV-1-specific cellular immune responses will be determined:

- Primarily, IFN-у ELISPOT responses using fresh PBMC including determination of cross-clade responses, using pools of overlapping peptides representing the immunogens.
- 8-colour intracellular cytokine staining (ICS) using fresh PBMCs and conducted against the same peptide pools as in the ELISPOT and testing both for CD8 and CD4 T cell responses.
- Multicolour ICS staining for measuring polyfunctional responses using cryopreserved PBMC, including cytokines/chemokines, markers for cytolytic capacity and immunphenotyping of responding cells.
- Mapping of novel epitopes recognised by vaccinees using matrices of peptides in ELISPOT.
- Lymphocyte proliferation assay (LPA) to immunizing antigens using fresh PBMC and a standard ^3^H-thymidine uptake assay and /or a flow cytometry based assay (CFSE or FASCIA).

Optional assay

- Viral inhibition assay
- Cellular reactivity against vaccinia antigens

*4.2.3 HLA analysis and immune reactivity gene sequences*

HLA typing will be performed by molecular methods at one time point. Analyses of immune reactivity gene sequences will optionally be performed. DNA is extracted by validated methodology from peripheral lymphocytes according to protocol. The DNA is stored at -20ºC under single code labelling and typed. The HLA type will be determined to oligo-allelic level by sequencing for classes I and II (HLA-A, -B, -C, DQ, -DR, -DPA1 and -DP1). Typing will then be performed by sequence-specific primers SSP-PCR (Olerup, GenoVision). HLA class I restricted alleles will be matched to epitopes giving rise to known or novel identified epitopes by a peptide-matrix analysis. The strength or increased strength of immune reactions (see above) will be compared to initial reactivities and to those of individuals receiving different doses of HIV DNA vaccine or receiving HIV MVA-CMDR. For class II reactivities the comparisons will be made to development of both antibody and CD4+ antigen-specific reactivity. Clinical sensitivity is related to the strength of immune reactivity by electroporation delivery compared to mode of vaccine delivery. The immunological response in each individual can be related to the changes of his/her own reactivities over time. The patient can participate in the study without the HLA typing.

**4.3 Other assessments**

*4.3.1 Antibodies against Vaccinia*

Serum samples will be stored for analysis of binding and neutralizing antibodies to vaccinia.

*4.3.2 Analysis of peptide reactivity (optionally)*

Cellular samples for analysis of peptide reactivity compared to preclinical animal reactivities after electroporation (preferably 3-4 times during the study).

*4.3.3 Analysis of anti-DNA reactivity (optionally)*

- Extended analysis of DNA antibodies.
- Cellular samples for analysis of anti-DNA reactivity.

*4.3.4 Punch biopsy (optionally)*

The presence of DNA vaccine plasmids will be determined in a punch biopsy. Histopathology and an attempt to estimate HIV gene presence and expression will be made.

*4.3.5 Genital infection*

The assays will be conducted at the SWEDAQ certified Labmedicin Huddinge University Hospital laboratories if clinically indicated.

*4.3.6 Following adverse event*

Other assessments may be performed as clinically indicated due to an adverse event.

*4.3.7 Concomitant medication*

Participants will be asked about medication taken at each visit up to and including week 60, and this will be recorded in the case record form. Thereafter, they will be asked about medication only in relation to adverse events reported. It is expected that the name of the drug, indication for use, dose, frequency, start and stop dates will be available for prescription-only medication, either from the participant or from the prescribing physician. For medication available over the counter, the maximum information available on questioning the participant will be recorded.

### 5 Adverse events

Participants will be provided with a diary card and disposable thermometers at the immunizations. A study physician or nurse will be on call 24 hours a day for the duration of the study. All participants will be provided with a direct telephone number to call in case of any suspected adverse event.

**5.1 Definitions**

An adverse event is any adverse experience occurring during the course of the study including the screening period.

Criteria for grading clinical and laboratory events are listed in the appendix.

The severity of events reported on the adverse event form will be determined by the investigator, based on the Toxicity Grading Scale or if the event is not listed the event will be graded following these guidelines:

Mild (Grade 1): Transient or mild discomfort. No limitation in normal daily activity.

Moderate (Grade 2): Some limitation in normal daily activity.

Severe (Grade 3): Unable to perform normal daily activity.

Serious (Grade 4): Life threatening

Death (Grade 5)

A severe adverse event is one graded 3 or 4 by criteria in appendix. Some, but not all grade 3 and 4 adverse events will be “serious” by ICH GCP criteria below.

*5.1.1 Serious Adverse Events (SAEs)*

An adverse event is considered to be a “serious adverse event” by ICH Good Clinical Practice (ICH GCP) criteria if it results in the following:

- death,
- a threat to life,
- requires in-patient hospitalisation or prolongs existing hospitalisation (hospitalisation for elective treatment of a pre-existing condition is not included),
- results in persistent or significant disability or incapacity,
- is a congenital anomaly (i.e., the outcome of pregnancy involving a participant), or
- is any other important medical condition*.

*Examples of conditions regarded as “any other important medical condition” include allergic bronchospasm requiring intensive emergency treatment, seizures or blood dyscrasias which did not result in hospitalisation or development of drug dependency.

**5.2 Relationship to study product**

This can be classified as:

**None** adverse events felt to be due to extraneous causes that neither follow a known pattern of response nor a reasonable temporal relationship to study product

**Remotely related** adverse events that are unlikely to be related to product but which follow a reasonable temporal relationship, such that this cannot be completely excluded or events that could be associated with product but which are unrelated in time

**Possibly related** adverse events that may be due to extraneous causes but which follow a known pattern of response and/or a reasonable temporal relationship to study product

**Probably related** adverse events that cannot be explained by extraneous causes; which follow a know pattern of response and/or a reasonable temporal relationship; which disappear or decrease on cessation of study product and reappear on re-challenge

**5.3 Reporting adverse events**

Adverse events should be recorded on the appropriate case record form as to the nature of the event, the time it started and ended, the severity, probable relationship to the drug and any medication taken.

Grade 2 events probably or possible related to the vaccine should be discussed in the weekly Trial Management Group meetings.

Any grade 3 or 4 adverse event remotely, possibly or probably related to the immunization should immediately be reported to the PI or his deputy, who will fill in the appropriate forms and decide discontinuation of the vaccination schedule. The PI should if indicated arrange for urgent review of the case by the Steering Committee and report this to the monitor and the DSMC within 2 working days. The monitor will confirm that the event is potentially reportable and inform the Sponsor. Should the case be considered reportable by the DSMC, a report will be prepared by the clinical investigator or his deputy and filed with the Sponsor, Medical Products Agency and the Regional Ethics Committee.

**SAEs** should be reported to the monitor and the DSMC the same working day that the Clinical Investigator becomes aware of the event. This can be done by telephone or fax. The minimum criteria required in reporting a SAE are the participant identifiers (trial number/ date of birth/initials), reporting source (name of Investigator), and why the adverse event is identifiable as serious.

**The adverse event reporting:**

**telephone/fax number 08- 511 792 66,**

**mobile number 070-44 25 770**

The PI arranges for urgent review of the case by the Steering Committee within 2 working days. The monitor will confirm that the event is potentially reportable and inform the sponsor.

Should the case be considered reportable by the DSMC, a report will be prepared by the clinical investigator or his deputy and filed with the Sponsor, Medical Products Agency and Regional Ethics Committee.

Examples of SAEs that do not require expedited reporting include:

- Hospitalisation for scheduled surgery unrelated to vaccine, other than temporally (within 30 days)
- Orthopaedic or traumatic injuries requiring hospitalisation
- Hospitalisation planned for pre-existing conditions not due to an aggravation in the condition
- SAEs occurring more than 30 days after vaccination or having appeared before vaccination without any aggravation after vaccination

**5.4 Clinical management**

Events will be managed by the clinical trial team who will assess and treat the event as appropriate.

**6 Management of HIV issues during and following the trial**

Individuals who test HIV-positive during the screening process will be offered care according to Swedish national guidelines at Venhälsan or referred to a care giver of their choice.

**6.1 HIV testing**

Only volunteers with a negative HIV antibody/antigen test result will be enrolled. A risk assessment will be undertaken prior to every immunisation and if risk status has changed then immunisation may have to be delayed until the HIV status of the participant is clarified. It is possible that participants may develop antibodies and test ‘positive’ in routine HIV ELISA assays subsequent to immunisation. The immunological and virological laboratories at SMI, will conduct additional tests to distinguish between infection and a post-immunisation response required either for clinical management, or at the request of a participant. The results from these tests will only be known to the study staff as positive or negative for HIV infection. At the end of the study specimens will be tested in real-time so that participants can be informed of the result and a plan to recall and retest them made should this be necessary. In the event of ongoing post-immunisation positive ELISA, the participants will be invited to re-attend annually until such time as this response has disappeared, and provided with an explanatory identity card in the interim.

*6.1.2 Verification of HIV status of participants*

If certification is required at the request of the participant, this can be provided by the trial team after testing at the local laboratory.

Results will always be given at an interview with a member of the study team unless the participant requests an independent physician, in which case this will be arranged.

If a specimen from a participant suggests that the participant is HIV infected, a second specimen will be collected and retested.

If a subject for any reason needs to establish his or her HIV status extended tests will be offered i.e. HIV RNA PCR or antigen test.

**6.2 HIV infection**

In the unexpected circumstances that a participant in the trial acquires HIV infection, they will be managed in the following way:

*6.2.1 Referral for clinical care*

Participants will be referred initially to a specialist physician at Venhälsan for a full discussion of the clinical aspects of HIV infection and planning for further care. Further investigations will be undertaken as necessary.

*6.2.2 Referral for counselling:*

A referral to a counsellor at Venhälsan will be arranged by the specialist physician. The counselling process will assist the participant in the following issues:

1. psychological and social implications of HIV infection
2. who to inform and what to say
3. implications for sexual partners
4. avoidance of risk to others in future

*6.2.3 Informing the other involved health care personnel and dentist*

The participant will be encouraged to do this, but the decision will remain at the discretion of the individual.

*6.2.4 Immunological follow-up*

Follow-up of HIV infected individuals who have received study vaccine products will be determined by the Trial Management Group. The intensity of assessments will be dependent both on the number of immunisations received by the individual and their clinical progress, including changes in disease markers such as viral load and CD4 count.

If a volunteer is found to be HIV-infected during the trial, he/she will be excluded from further immunizations in the trial.

**6.3 Social discrimination as a result of a post-vaccine response**

The aim is to minimise the possibility of social discrimination in participants who develop a positive HIV-ELISA test by providing HIV testing by PCR and certification for participants as required. In addition, an identification card stating that the individual has participated in a vaccine trial, with a contact number in case of medical emergency, will be provided.

In the unlikely event that a participant suffers social discrimination as a result of a post-vaccination response, the clinical investigators will assist the participant.

**7 Management of the trial**

**7.1 Data management at the Clinical Centre**

All procedures will be performed according to GCP. Data will be recorded directly onto the case report forms, which will provide the bulk of the data for the trial. The volunteer will only be identified by the study number and initials in the CRF. There will be additional confirmatory and source data in the clinical notes, such as medical history related to eligibility, results of laboratory analyses, dates of visits including immunisation, and details of clinical management (description of adverse events and concomitant medication). One photocopy of the completed case report form will be forwarded to the trial coordination centre for inspection and entry into the database.

Any changes to the CRFs should be done without obscuring the original entry, signed and dated, including changes made before a copy is forwarded to the trial coordination centre for inspection and data entry.

Study related documents should be kept in a secure location at least 15 years after completion of the trial. Clinical notes and original laboratory results should be kept according to applicable laws.

**7.2 Data management in the virology/immunology laboratories**

Standardised operating procedures according to GCLP will be followed in all laboratories to ensure the quality of the data. Data will be stored electronically in an agreed format and data files transferred to the trial coordinating centre for the main analysis.

**7.3 Data management at the trial coordination centre**

All CRFs will be reviewed for completeness and inconsistencies by the data manager according to standardised operating procedures. Any enquires will be communicated with the clinical centre and will be passed for review by the trial physician if required. The data manager will approve the CRFs for data entry into the data base. Data will be coded and entered into a computerised database. Consistency checks and range checks will be performed at the data entry level.

The patient database Melior will be used under the same security arrangements as for patient records.

After approval and entry into the database, the copy including documentation detailing all the queries and changes will be stored in a study specific binder at the trial coordination centre. The documents in the binder will be organised due to study number. The original case report forms will be stored together with the individual source data at the clinical centre.

**7.4 Monitoring**

The monitor will be recruited from Karolinska Trial Alliance, a Swedish study-independent centre with GCP educated professional staff specialized in study-associated services.

Karolinska Trial Alliance (KTA) Hälsingegatan 43, 17176 Stockholm

http://www.karolinskatrialalliance.se/

The monitor will be responsible for:

- Monitoring the trial according to ICH GCP guidelines including monitoring vaccine accountability, and dispatch and arrival of virological and immunological specimens
- Preparation of reports to assist the monitoring

The monitor will visit the clinical center to validate trial data against the clinical records. The monitoring will adhere to ICH Good Clinical Practice guidelines. The following data should be verifiable from source documents:

- completeness of the study file including all approvals, CVs etc
- documentation of any existing conditions or past conditions relevant to eligibility
- signed consent
- dates of visits including dates of immunisations
- source verification of all laboratory data which are out of the normal range entered into the data base
- source verification of all grade 2 or above adverse events entered into the data base

and any events leading to discontinuation of the immunisation schedule

Vaccine returns will also be monitored at visits to the clinical centre/pharmacy*.*

**7.5 Data Ownership**

The data generated in this study will be the property of SMI and made available to the Steering Committee for analysis and publication. If required at an earlier date for the purposes of regulatory submissions, a request should be made to the Trial Coordinating Committee.

- 1. **Trial committees**

*7.6.1 Steering Committee (SC)*

The supervision of the trial will be a responsibility of the Steering Committee (SC). It will also be charged with the compilation of data, analysis and final reports from the study. The committee will have an independent chair, Ragnar Norrby. The members will include Professor Britta Wahren, Professor Gunnel Biberfeld, Professor Eric Sandström, Associated professor Sören Andersson, Dr Mary Marovic and Dr Bo Hejdeman or their deputies.

Additional non-voting members will attend as appropriate to the contents of the meeting. This committee will be responsible for final decisions about grade of adverse events and relationship to study vaccine. The Steering Committee may form an endpoint committee to review the grade of adverse events and relationship to study vaccine, and the independent members will have an additional casting vote should this be required. Notes of meetings will be kept.

The trial may be terminated by this Committee for any reason, including on the recommendation of the DSMC.

*7.6.2 Trial Management Group (TMG)*

This group will oversee the day to day running of the trial and the members will be primarily related to the clinical and data management teams. The immunologists will join if there are relevant items on the agenda. Notes will be taken and will form the basis of the progress report to the Trial Coordinating Committee.

- 1. **Data and Safety Monitoring Committee (DSMC)**

An independent Data and Safety Monitoring Committee (DSMC) will be established by the sponsor to assess at intervals the progress of the clinical trial, the safety data, and the endpoints, and to recommend to the sponsor whether to continue, modify, or stop a trial. In the case of voting, the chairman will have the final say. The DSMC will meet in order to review the safety data after half of the participants have completed the full immunization regimen. Written reports of their meetings will be submitted to the Sponsor and the SC. The members of this DSMC are:

Peter Liljeström Chair

Patrick Olin Clinician

Anders Sönnerborg Clinician

*7.7.1 Indications for additional reviews*

There will be an additional meeting if any of the participants experience any grade 3 or 4 clinical or laboratory event (confirmed on attendance or repeat testing) not resolved within 72 hours and considered probably or possible and likely to be related to vaccine product or any reportable SAE. The DSMC will also be consulted in case of any event that is unexplained or unexpected that could pose a possible threat to the safety of the participants, SUSAR. The DSMC may also meet and ask for safety or efficacy data at their own discretion for any reason of their own.

*7.7.2 Interim analysis*

An interim analysis will be performed for safety when 10 volunteers have received the first electroporation and when half of the volunteers have received the last DNA injection.

*7.7.3 Indications for discontinuation of immunisations in all participants*

See section *3.7.2* above

*7.7.4 Analysis*

An analysis plan will be submitted to the DSMC before the code is broken.

### 8 Statistical considerations

**8.1 Sample size**

Safety: By the end of this study an additional (compared to previous clinical trials) 40 participants will have been exposed to HIV DNA and 30 the HIV MVA. In addition 36 individuals will have been exposed to 3 electroporation procedures and will be described in the qualitative safety evaluation. This is considered sufficient for the planning of continued studies.

Immunology, secondary endpoints: The size of the groups (10+2) and the comparisons described below, 8.2, is judged to be sufficient in a descriptive study in order to formulate the hypothesis to be tested in a planned trial in Tanzania.

**8.2 Analysis**

All safety end-points will be graded by the Clinical Investigator and reviewed by the Trial Management Group. Any queries about grade and relationship to study product that cannot be resolved will be referred to the Trial Coordinating Committee for a final decision.

The primary end-points:

1. The safety of immunization with the seven or eleven DNA plasmids carrying HIV-1 genes will be assessed by clinical signs and standard biochemical and haematological laboratory tests where any worsening of the severity grade will be considered for causality with the vaccine.
2. The immunization will be evaluated by assessing local (pain, cutaneous reactions including induration), general (fever, chills, headache, nausea, vomiting, malaise, myalgia) and other unsolicited adverse events within 7 and 28 days. Any grade 3 or 4 event will be taken as an indication that the arm is less tolerable.

The secondary endpoints

The secondary endpoint (immunogenicity) will primarily be assessed by IFN-γ ELISpot 2 weeks after the third DNA immunization, the fourth DNA or first MVA immunization and after the fifth DNA or second MVA immunization. Both number of responders and magnitude of responses will be tested for significance. Additional studies will be performed at other time points using other assays such as intracellular cytokine staining, lymphoproliferation assays, epitope mapping, binding (and if indicated) neutralization antibody assays.

Group B1 will be compared with the id only group in the HIVIS study (Group A in HIVIS trial) for evaluation of the lower dose of HIV DNA (0.6 vs 1.0 mg).

Group B2 will be compared with Group B1 for evaluation of the lower dose of HIV DNA with and without electroporation.

Group B3 will be compared to the group B2 for the evaluation of HIV DNA as a boost compared to boosting with HIV MVA.

Group B4 is will be compared to groups B1-B3 to show if priming with additional HIV-related plasmids will increase immunogenicity to any of the included antigens.

Figures/tables will be prepared including all participants’ results.

### 9 Confidentiality, ethics and responsibilities, including indemnity

Full medical confidentiality will be preserved.

The study will be conducted according to ICH GCP guidelines and the Declaration of Helsinki (version 2008), and is the responsibility of the Clinical Investigators.

The Sponsor has delegated the following responsibilities as outlined below.

The Principal and Clinical Investigators are responsible for obtaining the appropriate Regional Ethics Committee approval for the study protocol, the subject information sheet and the consent form. The Principal and Clinical Investigators are responsible for informing the ethics committee of any SAEs as required, and submitting annual reports as required.

Regulatory submissions will be made in Sweden, in collaboration with Professor Wahren and a regulatory consultant. Professor Wahren will take responsibility for providing required documentation for the HIV DNA vaccines, the Walter Reed Army Institute of Research for the HIV MVA vaccine. Regulatory approval must be provided before study materials will be shipped to the clinical centre. Vecura will be responsible for supply and labelling of the DNA.

The SC will be responsible for assigning an independent statistician to prepare the randomisation list, all aspects of data management including preparing for study-independent monitoring of the clinical sites, and the analysis. Staff will also be responsible for coordinating the response to any SAEs that arise during the course of the trial and reporting these if indicated to the regulatory authorities.

The Swedish Institute for Infectious Disease Control will act as Sponsor for the trial, and will coordinate the necessary clinical trial agreements delineating the above responsibilities and the liability for events occurring as a result of participating in the trial. These agreements must be signed by all parties involved before materials are shipped to the relevant clinical centre. Indemnity will be ensured by the following parties:

- The Principal Investigators will ensure that all clinical staff engaged in the study are covered for negligent harm to a participant, either through a personal or hospital insurance scheme
- Vecura will ensure that a policy providing similar assurance for the DNA vectors is in place. The pharmaceutical insurance will indemnify those suffering adverse events of insured pharmaceuticals if the injury with proponent probability has been caused by a pharmaceutical.
- The Swedish Institute for Infectious Disease Control will be responsible as Sponsor for ensuring that a policy is in place to cover ‘no fault compensation’ for participants on the trial who suffer an adverse event that is attributable to participation in the trial but which is not related to product or due to negligent harm caused by the clinical staff.

Volunteers, as well as patients, in clinical trials performed in the health care settings and approved by an ethics committee are by Swedish law indemnified for damage due to negligent harm caused by the clinical staff.

# 10 Protocol amendments

The protocol team prepares any amendments needed for approval of the HIVIS steering committee. Amendments to the protocol will be made only after consultation and agreement between sponsors and investigator. The only exception is where the investigator considers that a subject’s safety is compromised without immediate action. All amendments that have an impact on subject risk or the study objectives, or require revision of the informed consent document, must receive approval from the Medical Products Agency (Läkemedelsverket) and the ethical committee prior to their implementation. Relevant information in the investigators brochure (IB, prövarhandbok) and participant information (deltagarinformation) will be updated in the event of submission of an amendment to the protocol and submitted to MPA.

**11 Publication**

It is intended that the results of this study will be published in an appropriate peer-reviewed journal, with the aim of submitting a paper for publication within 6 months of the study’s completion. The HIVIS Steering Committee and the HIVIS Trial Coordinating Committee including Bioject Inc and Cyto Pulse Sciences Inc will have 30 days to comment on any manuscript. No other publications, whether in writing or verbally, will be made before the definitive manuscript or abstract for a scientific meeting has been agreed and accepted for publication, without the prior approval of these committees.

A final report of the study will be prepared by the investigators and circulated to the HIVIS Steering Committee for comments.

**12 References**

Bakari M,F Mhalu, S Aboud, C Nilsson, J Francis, M Janabi, E Lyamuya, EA Aris, J Mbwana, D Buma, L Mwanyika, B Hejdeman, A Bråve, M Robb, M Marovich, N Michael, P Earl, B Wahren, G Biberfeld, K Pallangyo, E Sandström, for the HIVIS study group. Safety and immunogenicity of an HIV-1 DNA plasmid vaccine boosted with HIV-1 MVA among Police Officers (PO’s) in Dar es Salaam, Tanzania (HIVIS03). AIDS vaccine 2009, Paris 2009

Bakari M, F Mhalu, S Aboud, C Nilsson, J Francis, M Janabi, E Lyamuya, EA Aris, J Mbwana, D Buma, L Mwanyika, B Hejdeman, A Bråve, M Robb, M Marovich, N Michael, P Earl, B Moss, B Wahren, G Biberfeld, K Pallangyo, E Sandström, for the HIVIS study group. Safety and immunogenicity of a multigene multiclade HIV-1 DNA plasmid vaccine boosted with HIV-1 MVA among healthy volunteers in Dar es Salaam, Tanzania. AIDS vaccine 2008, Cape Town 2008

Boberg A, Bråve A, Johansson S, Wahren B, Hinkula J, Rollman E. [Murine models for HIV vaccination and challenge.](http://www.ncbi.nlm.nih.gov/pubmed/18251698?ordinalpos=10&itool=EntrezSystem2.PEntrez.Pubmed.Pubmed_ResultsPanel.Pubmed_DefaultReportPanel.Pubmed_RVDocSum) Expert Rev Vaccines.7:117-30. Review. 2008

Bråve A, Wahren B. Experimental and clinical approaches for genetic immunization against HIV-1 DNA vaccines, Las Vegas Dec 2008.

Calarota SA, Leandersson AC, Bratt G, Hinkula J, Klinman DM, Weinhold KJ, Sandström E, Wahren B. [Immune responses in asymptomatic HIV-1-infected patients after HIV-DNA immunization followed by highly active antiretroviral treatment.](http://www.ncbi.nlm.nih.gov/pubmed/10438897?ordinalpos=9&itool=EntrezSystem2.PEntrez.Pubmed.Pubmed_ResultsPanel.Pubmed_DefaultReportPanel.Pubmed_RVDocSum) J Immunol. 15;163(4):2330-8, 1999

Calarota S, Bratt G, Nordlund S, Hinkula J, Leandersson AC, Sandström E, Wahren B. [Cellular cytotoxic response induced by DNA vaccination in HIV-1-infected patients.](http://www.ncbi.nlm.nih.gov/pubmed/9643795?ordinalpos=6&itool=EntrezSystem2.PEntrez.Pubmed.Pubmed_ResultsPanel.Pubmed_DefaultReportPanel.Pubmed_RVDocSum) Lancet. 1998 May 2;351(9112):1320-5

Cristillo AD, Weiss D, Hudacik L, Restrepo S, Galmin L, Suschak J, Draghia-Akli R, Markham P, Pal R. [Persistent antibody and T cell responses induced by HIV-1 DNA vaccine delivered by electroporation.](http://www.ncbi.nlm.nih.gov/pubmed/18036339?ordinalpos=17&itool=EntrezSystem2.PEntrez.Pubmed.Pubmed_ResultsPanel.Pubmed_DefaultReportPanel.Pubmed_RVDocSum) Biochem Biophys Res Commun. 366(1):29-35, 2008

CytoPulse http://www.cytopulse.com/electroporation.shtml

Dorrell L, Williams P, Suttill A, Brown D, Roberts J, Conlon C, et al. Safety and tolerability of recombinant modified vaccinia virus Ankara expressing an HIV-1 gag/multiepitope immunogen (MVA.HIVA) in HIV-1-infected persons receiving combination antiretroviral therapy. Vaccine; 25(17):3277-83. 2007

Hirao LA, Wu L, Khan AS, Satishchandran A, Draghia-Akli R, Weiner DB. [Intradermal/subcutaneous immunization by electroporation improves plasmid vaccine delivery and potency in pigs and rhesus macaques.](http://www.ncbi.nlm.nih.gov/pubmed/18082294?ordinalpos=15&itool=EntrezSystem2.PEntrez.Pubmed.Pubmed_ResultsPanel.Pubmed_DefaultReportPanel.Pubmed_RVDocSum) Vaccine. 26(3):440-8, 2007

Hirao LA, Wu L, Khan AS, Hokey DA, Yan J, Dai A, Betts MR, Draghia-Akli R, Weiner DB. [Combined effects of IL-12 and electroporation enhances the potency of DNA vaccination in macaques.](http://www.ncbi.nlm.nih.gov/pubmed/18430495?ordinalpos=11&itool=EntrezSystem2.PEntrez.Pubmed.Pubmed_ResultsPanel.Pubmed_DefaultReportPanel.Pubmed_RVDocSum) Vaccine. 26(25):3112-20, 2008

Jaoko W, Nakwagala FN, Anzala O, Manyonyi GO, Birungi J, Nanvubya A, et al. Safety and immunogenicity of recombinant low-dosage HIV-1 A vaccine candidates vectored by plasmid pTHr DNA or modified vaccinia virus Ankara (MVA) in humans in East Africa. Vaccine (22):2788-95, 2008

Kiepiela P, Ngumbela K, Thobakgale C, Ramduth D, Honeyborne I, Moodley E, et al. CD8+ T-cell responses to different HIV proteins have discordant associations with viral load. Nat Med 2007 Jan;13(1):46-53.

Leandersson AC, Gilljam G, Fredriksson M, Hinkula J, Alaeus A, Lidman K, Albert J, Bratt G, Sandström E, Wahren B. [Cross-reactive T-helper responses in patients infected with different subtypes of human immunodeficiency virus type 1.](http://www.ncbi.nlm.nih.gov/pubmed/10775629?ordinalpos=8&itool=EntrezSystem2.PEntrez.Pubmed.Pubmed_ResultsPanel.Pubmed_DefaultReportPanel.Pubmed_RVDocSum) J Virol. 74(10):4888-90, 2000

Luckay A, Sidhu MK, Kjeken R, Megati S, Chong SY, Roopchand V, Garcia-Hand D, Abdullah R, Braun R, Montefiori DC, Rosati M, Felber BK, Pavlakis GN, Mathiesen I, Israel ZR, Eldridge JH, Egan MA. [Effect of plasmid DNA vaccine design and in vivo electroporation on the resulting vaccine-specific immune responses in rhesus macaques.](http://www.ncbi.nlm.nih.gov/pubmed/17329330?ordinalpos=20&itool=EntrezSystem2.PEntrez.Pubmed.Pubmed_ResultsPanel.Pubmed_DefaultReportPanel.Pubmed_RVDocSum) J Virol. 81(10):5257-69, 2007

Lundholm, P., Leandersson, A.C., Christensson, B., Bratt, G., Sandström, E. & Wahren, B.: DNA mucosal HIV vaccine in humans. Virus Res., 82:1-2; 141-145, 2002

Moorthy VS, McConkey S, Roberts M, Gothard P, Arulanantham N, Degano P, et al. Safety of DNA and modified vaccinia virus Ankara vaccines against liver-stage P. falciparum malaria in non-immune volunteers. Vaccine; 21:17-18. 2003

Mäkitalo B, Lundholm P, Hinkula J et al. Enhanced cellular immunity and systemic control of SHIV infection by combined parenteral and mucosal adminstration of a DNA prime MVA boost vaccine regimen. J Gen Virol; 85: 2407-2419. 2004

National AIDS Control Programme, United republic of Tanzania. HIV/AIDS/STI Surveillance report. 16:2001.

Peters BS, Jaoko W, Vardas E, Panayotakopoulos G, Fast P, Schmidt C, et al. Studies of a prophylactic HIV-1 vaccine candidate based on modified vaccinia virus Ankara (MVA) with and without DNA priming: effects of dosage and route on safety and immunogenicity. Vaccine (11):2120-7. 2007

Rerks-Ngarm S, Pitisuttithum P, Nitayaphan S, Kaewkungwal J, Chiu J, Paris R, et al. Vaccination with ALVAC and AIDSVAX to prevent HIV-1 infection in Thailand. N Engl J Med 2009; 23:2209-20.

Roos AK, Moreno S, Leder C, Pavlenko M, King A, Pisa P. [Enhancement of cellular immune response to a prostate cancer DNA vaccine by intradermal electroporation.](http://www.ncbi.nlm.nih.gov/pubmed/16185933?ordinalpos=6&itool=EntrezSystem2.PEntrez.Pubmed.Pubmed_ResultsPanel.Pubmed_DefaultReportPanel.Pubmed_RVDocSum) Mol Ther. 13(2):320-7, 2006

Roos AK, Eriksson F, Timmons JA, Gerhardt J, Nyman U, Gudmundsdotter L, Bråve A, Wahren B, Pisa P. Skin electroporation: effects on transgene expression, DNA persistence and local tissue environment. PLoS One. 4(9):7226, 2009

Sandström E, Nilsson C, Hejdeman B, Bråve A, Bratt G, Robb M, Cox J et al. B[road immunogenicity of a multigene, multiclade HIV-1 DNA vaccine boosted with heterologous HIV-1 recombinant modified vaccinia virus Ankara.](http://www.ncbi.nlm.nih.gov/pubmed/18808335?ordinalpos=1&itool=EntrezSystem2.PEntrez.Pubmed.Pubmed_ResultsPanel.Pubmed_DefaultReportPanel.Pubmed_RVDocSum) J Infect Dis. 198:1482-90, 2008

UNAIDS/WHO 2003. AIDS epidemic update. Dec 2008.

Vasan S, In vivo electroporation enhances the immunogenicity of ADVAX, a DNA-based HIV-1 vaccine candidate, in healthy volunteers. AIDS vaccine 2009, Paris 2009
