# Supplementary material for: HIV-DNA Given with or without Intradermal Electroporation Is Safe and Highly Immunogenic in Healthy Swedish HIV-1 DNA/MVA Vaccinees: A Phase I Randomized Trial
Source: PLoS One. 2015 Jun 29;10(6):e0131748. doi: 10.1371/journal.pone.0131748 (PMC4486388; doi:10.1371/journal.pone.0131748)
Supplement: S1 Table — (DOCX) [file pone.0131748.s004.docx]

**Table S1. IFN-γ ELISpot response rates after three HIV-DNA immunizations.**

| Peptide pool | Immunization group (no, %^a^) | | |
| --- | --- | --- | --- |
|  | Group 1 | Group 2 | Group 3 |
| Gag I (p17) ^b^ | 1/7 (14%) | 1/7 (14%) | 3/8 (37%) |
| Gag II (p24) ^b^ | 1/7 (14%) | 2/7 (28%) | 1/8 (12%) |
| Gag CMDR | 1/6 (17%) | 2/7 (28%) | 1/7 (14%) |
| Env I (gp120 V1-V2) ^b^ | 1/7 (14%) | 0/7 | 0/8 |
| Env II (gp120 V3-V5) ^b^ | 0/7 | 0/7 | 0/8 |
| Env III (gp41) ^b^ | 0/7 | 0/7 | 0/8 |
| Env CMDR | 0/7 | 0/7 | 0/8 |
| Pol CMDR | 0/7 | 0/7 | 0/8 |
| Any Gag | 1/7 (14%) | 2/7 (28%) | 3/8 (37%) |
| Any Env | 1/7 (14%) | 0/7 | 0/8 |
| Gag or Env | 1/7 (14%) | 2/7 (28%) | 3/8 (37%) |

^a^Frequency of responders given as percentage of total number of evaluable vaccinees. ^b^HIV-DNA-vaccine subtype A- and B-specific peptides. All peptides were 15-mers with 10-aa overlap. The protein specificity is indicated in parentheses. The peptides included in Gag CMDR, Env CMDR and Pol CMDR were specific for the HIV inserts in MVA and were 15-mers with 11-aa overlap.
