# Supplementary material for: HIV-DNA Given with or without Intradermal Electroporation Is Safe and Highly Immunogenic in Healthy Swedish HIV-1 DNA/MVA Vaccinees: A Phase I Randomized Trial
Source: PLoS One. 2015 Jun 29;10(6):e0131748. doi: 10.1371/journal.pone.0131748 (PMC4486388; doi:10.1371/journal.pone.0131748)
Supplement: S2 Table — (DOCX) [file pone.0131748.s005.docx]

**Table S2. IFN-γ ELISpot response rates to HIV-DNA-specific peptide pools.**

| Peptide pool | Two weeks after 1^st^ HIV-MVA  boost, n=19 | | | Two weeks after 2^nd^ HIV-MVA boost, n=15 | | |
| --- | --- | --- | --- | --- | --- | --- |
|  | Immunization group (no, %^a^) | | | Immunization group (no, %^a^) | | |
|  | Group 1 | Group 2 | Group 3 | Group 1 | Group 2 | Group 3 |
| Gag I | 5/6 (83%) | 5/7 (71%) | 4/6 (67%) | 5/6 (83%) | 4/5 (80%) | 3/4 (75%) |
| Gag II | 6/6 (100%) | 7/7 (100%) | 5/6 (83%) | 4/6 (67%) | 4/5 (80%) | 4/4 (100%) |
| Env I | 5/6 (83%) | 2/7 (29%) | 3/6 (50%) | 2/6 (34%) | 0/5 | 2/4 (50%) |
| Env II | 1/6 (17%) | 1/7 (14%) | 1/6 (17%) | 0/6 | 0/5 | 1/4 (25%) |
| Env III | 2/6 (34%) | 3/7 (43%) | 3/6 (50%) | 1/6 (17%) | 0/5 | 2/4 (50%) |

^a^Frequency of responders given as percentage of total number of vaccinees.
